# Broadband dispersion-engineered Bragg grating mirrors for integrated side-coupled Fabry–Pérot resonators

**DOI:** 10.1038/s41598-026-51041-9

**Published:** 2026-05-18

**Authors:** Davide Monopoli, S. Hadi Badri, Nicola Maraviglia, Artem S. Vorobev, Natale G. Pruiti, G. C. R. Devarapu, Marc Sorel, Giovanna Calò, Liam O’Faolain

**Affiliations:** 1 Centre for Advanced Photonics and Process Analysis, Munster Technological University, Cork, T12 P928 Ireland; 2 Tyndall National Institute, Cork, T12PX46 Ireland; 3 Department of Electrical and Information Engineering, Polytechnic University of Bari, Bari, 70126 Italy; 4 University of Glasgow, Glasgow, G12 8LT UK; 5 Institute of Technologies for Communication, Information and Perception (TeCIP), Sant’Anna School of Advanced Studies, Via Moruzzi 1, Pisa, 56127 Italy

**Keywords:** Optics and photonics, Physics

## Abstract

We present the design and demonstration of silicon nitride side-coupled Fabry–Pérot resonators employing both uniform and apodised distributed Bragg reflectors, for broadband dispersion engineering. By implementing air-gap Bragg gratings, we achieve a broad stopband exceeding 70 nm. Apodised gratings enable enhanced dispersion control, allowing transitions from normal to anomalous integrated dispersion. Furthermore, high-Q resonant modes and enhanced peak reflectivity are demonstrated through co-optimisation of cavity length and waveguide-to-cavity gap. The compact footprint, the enhanced mode selectivity and the spectral bandwidth control, make side-coupled Fabry-Pérot resonators a promising platform for resonant mirrors in hybrid on-chip lasers, as well as for sensing and non-linear photonic systems.

**Supplementary Information:**

The online version contains supplementary material available at 10.1038/s41598-026-51041-9.

## Introduction

Integrated photonic resonators are essential building blocks in the rapidly expanding field of photonic integrated circuits (PICs). Among these, traveling-wave resonators, such as micro-ring and whispering-gallery mode resonators have been extensively studied^1–3^.

While these systems are known for their high-quality factor (Q), their geometries impose limitations on the maximum free spectral range (FSR) achievable without encountering significant bending losses or added device complexity^4^. Moreover, at extremely high-Q (> 10^6^), random backscattering from surface roughness can induce mode-splitting of otherwise degenerate counter-propagating modes, posing additional challenges to increasing the Q-factor and overall reproducibility^5^. To address these limitations, standing-wave resonators, particularly Fabry-Pérot (FP) cavities, offer an efficient solution for achieving high-quality resonators while maintaining a compact footprint^6,7^. In FP cavities, light oscillates between two partially reflecting mirrors, typically realised as distributed Bragg reflectors (DBRs), forming a standing-wave pattern whose resonances are determined by the cavity length and the phase response of the mirrors.

The periodical variation of the waveguide effective index can be implemented through approaches ranging from cladding modulation^8^ to waveguide‑width corrugation^9,10^ to patterned features inside the waveguide, and full air‑gap gratings^11,12^. These implementations can provide precise control over mirror reflectivity, stopband width, group-delay dispersion, and their spectral response through parameters such as periodicity, duty cycle, and corrugation depth tailoring to specific application and fabrication constraints. Moreover, waveguide width modulation combined with apodised gratings enables fine tuning of the resonator operating bandwidth and dispersion profile^8^.

The compact, linear FP geometry allows for shorter cavity lengths, enabling single-resonance operation within the DBR stopband. FP resonators can employ either in-line or side-coupled (SC) configurations, depending on whether light accesses the cavity through one of the reflectors or via evanescent coupling from an adjacent waveguide.

Recent demonstrations of high-Q in-line FP resonators have significantly advanced the state of the art, enabling dispersive photonic functionalities^5^, non-linear applications^7,13^ and engineered-grating designs^6,14^.

In the side coupled configuration^4,15–18^, the access waveguide is decoupled from the resonant feedback path. As a result, scattering and radiation loss in the etched DBR sections occur inside the cavity and do not translate directly into additional insertion loss at the input/output ports. By adjusting the side coupler geometry, the external coupling can be optimised to minimise the impact of mirror losses on the bus waveguide, while the DBR mirror reflectivity can be designed independently. This decoupling between mirror and coupler design allows precise control of the coupling conditions and modal selectivity.

Accurate engineering of the photonic platform and reflector geometry can further enhance system stability and noise performance.

Implementing the resonators on a low-index-contrast platform such as silicon nitride (SiN) and operating at moderate group index renders the cavities intrinsically less susceptible to disorder- induced backscattering than devices on high-index-contrast SOI platforms^19^.

In addition, adiabatic cavity–reflector transitions at the DBR interfaces minimise mode mismatch and consequent radiation losses, further reducing sensitivity to fabrication-induced perturbations^14,20^. Moreover, the bidirectional propagation of the light in the SC-FPs cavity results in transmission dips and reflection peaks in the input and output section of the adjacent waveguide.

As for general cavities, the extinction ratio of the transmitted dips is governed by the interplay between internal losses and external coupling^21^.

In this work, we experimentally demonstrate SC-FP resonators on silicon nitride (SiN) platform by employing engineered DBR mirrors. We explored different duty cycles, and grating profiles, including air-gap structures^22^, to achieve operating DBR stopband exceeding 70 nm in the C-band. We show that control over the linearly apodised DBR corrugations together with the cavity length enable transition from normal to anomalous dispersion regimes, thereby supporting future applications in frequency comb generation^23^, and hybrid multi-wavelength laser systems^24^. For suitable choice of cavity length and coupling gap, the reflection spectra of the SC-FP exhibit well-defined peaks across the operating bandwidth, highlighting the potential of these devices as wavelength-selective resonant reflectors. These features are particularly attractive for hybrid integrated lasers, where fine control over cavity dispersion and resonance position is essential.

## Device concept and design

Our integrated SC-FPs, realised in SiN platform, consist of a straight waveguide forming a FP cavity terminating in two identical DBR sections. A third-order waveguided with a Bézier bend waveguide, positioned at a coupling gap (*G*), provides evanescent side-coupling to the cavity waveguide. A thermally oxidised bulk silicon wafer with a 300 nm thick SiN layer ($\:{t}_{SiN}$*)*, supplied by LioniX International BV, was used for the fabrication of the SC-FPs.

A 400 nm thick hydrogen silsesquioxane (HSQ) resist layer was deposited and patterned on top of the SiN film via electron-beam lithography (EBL), and the pattern was subsequently transferred into the SiN using inductively coupled plasma etching.

From the initial HSQ thickness, approximately 70 nm of residual mask material remained after etching.

This thin overlayer was deliberately left in place, as an additional HF-based wet-etch step would have exposed the SiO₂ lower cladding to HF, which could introduce extra scattering and absorption losses and compromise the integrity of the photonic structures.

The main fabrication steps are summarised in Fig. S1 in the Supplementary Material Sect. 1, while the presence of the residual HSQ and the resulting non-ideal, single-mode waveguide cross-section, attributed to a non-optimised dry-etch recipe, is confirmed by the scanning electron microscopy (SEM) inspection shown in Fig. S3 (Supplementary Material Sect. 1).

**Fig. 1:**
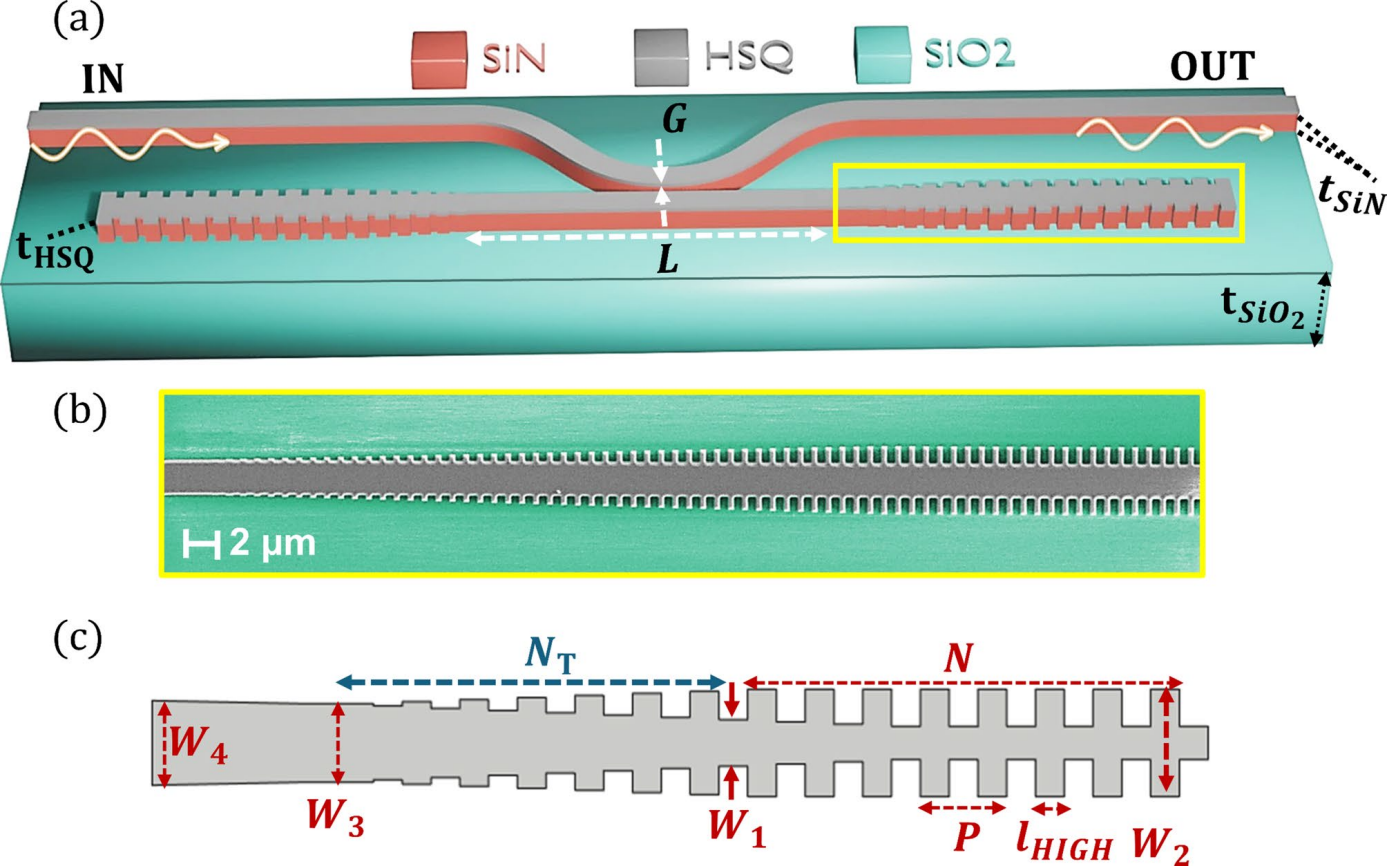
**a** Schematic of the integrated SC-FP with details on the layers thickness and coupling gap and cavity length parameters. **b** SEM image of the fabricated DBR: combination of uniform and apodised gratings. **c** Key design parameters of the implemented DBRs: by varying these values, different SC-FPs are obtained.

Figure 1a shows the schematic of the SC-FP incorporating uniform and apodised DBRs, whereas SEM inspection reported in Fig. 1(b) confirms the quality of the fabricated apodised grating.

The DBR is implemented by periodically modifying the waveguide width with a square wave modulation of the period *P*. The grating corrugations in the low- and high-index regions are engineered to enhance the effective index contrast, without entering a strongly multimode regime and simultaneously demonstrating the high fidelity of the etching process (as documented in *Fig. S2*,* S4*,* S5* in *Supplementary Material* Sect. 1*)*.

In the apodised version of the DBRs, the narrower and larger width of the modulation are linearly tapered from *W*_*3*_ to *W*_*1*_ and $\:{W}_{2}$, respectively, over the first *N*_*T*_ periods of the DBR as depicted in the schematic in Fig. 1(c).

At both edges of the cavity waveguide the DBR begins with the same width *W*_*3*_, where $\:{\mathrm{W}}_{{\mathrm{1}}} \le \:{\mathrm{W}}_{{\mathrm{3}}} \: < {\mathrm{W}}_{{\mathrm{2}}}$. Hereafter, if not specified, the nominal duty cycle (*DC*=$\:{\mathrm{l}}_{\mathrm{HIGH}}\mathrm{/P}$) of the DBR corrugation is *DC* = 50%.

For some devices specified in the text, to reduce sidewall scattering losses in the cavity without modifying the DBR section, the waveguide width in central part of the FP (*W*_*4*_) is increased so that *W*_*4*_ ≥ *W*_*3*_ and a short linear taper within the cavity connects this wider central section to the DBRs.

Furthermore, among the design parameters, we vary cavity length (*L*) and gap *G*, which along with the DBR parameters, control the coupling coefficient between the bus waveguide and the FP cavity, the SC-FP reflectivity, FSR, and dispersion as will be detailed in Sect. 3.

The implemented on-chip DBRs can be grouped in three distinct families (referred to as A, B, C), according to their mirror architecture, resulting in three corresponding SC-FP configurations.

**Fig. 2:**
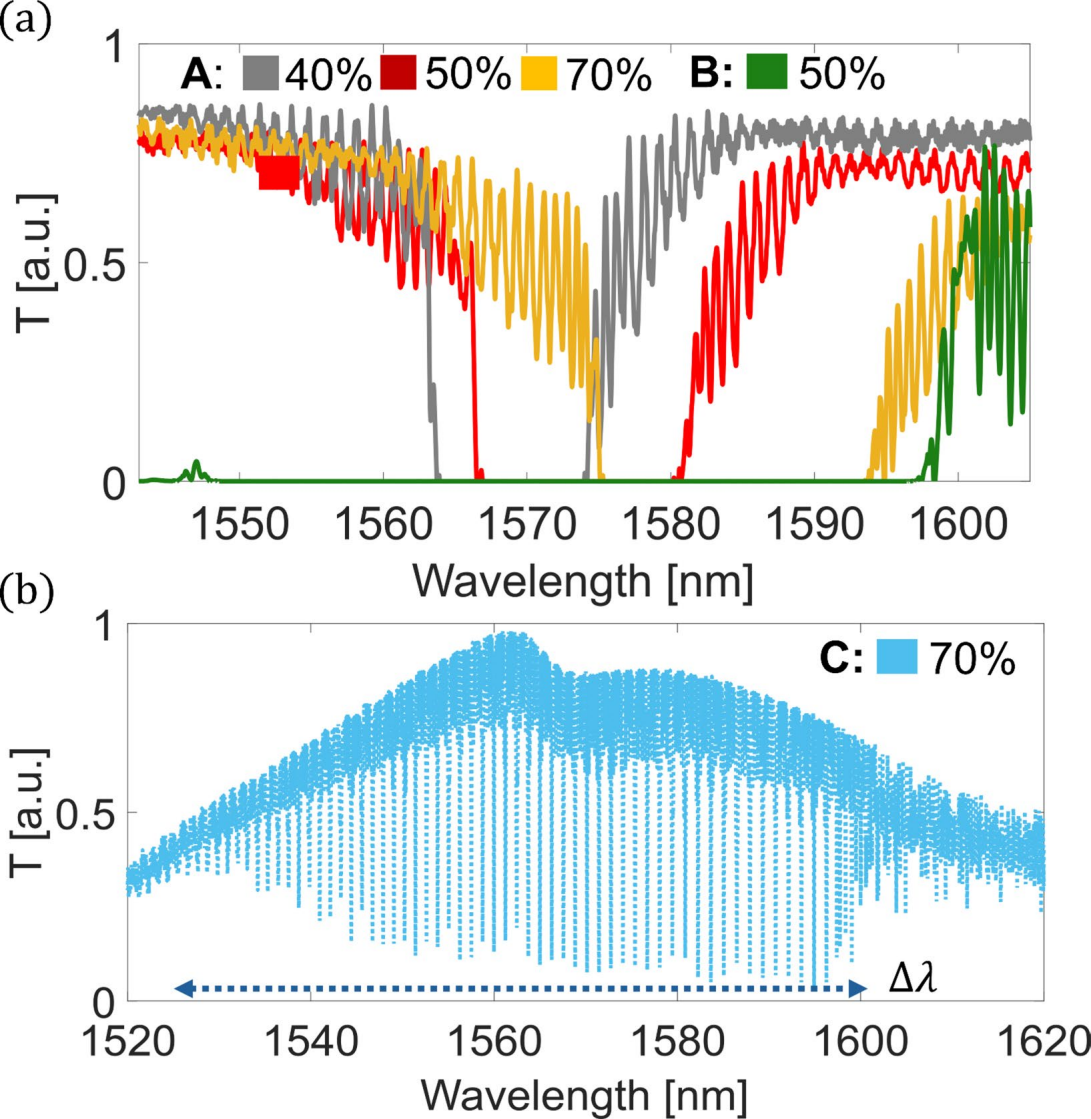
**a** Measured normalised transmission spectra for family A DBR with duty cycles of DC = 40%, 50%, and 70%, compared with family B architecture designed with DC = 50%. **b** Normalised transmission spectrum of air-gap SC-FP based on family C gratings (DC = 70%): a stopband Δ$\:\lambda\:$ ≈ 74 nm can be extracted.

Family A employs rectangular DBR corrugations with $\:{\mathrm{W}}_{{\mathrm{1}}} = \:{\mathrm{W}}_{{\mathrm{3}}} \:{\text{ = }}\:{\mathrm{1}}.{\mathrm{3}}\upmu {\mathrm{m}},$ and $\:{\mathrm{W}}_{{\mathrm{2}}} {\text{ = 2}}.{\mathrm{5}}\upmu {\mathrm{m}}$. Here, by varying the DC from 40% to 70%, the measured 3-dB stopband (Δ$\:\lambda\:$) increases from Δ$\:\lambda\:$ = 10.0 nm to Δ$\:\lambda\:$ = 18.8 nm, with a red-shift of the centre of the stopband position from 1569 nm to 1584 nm, for a fixed periodicity *P* = 506 nm (Fig. 2(a)).

Family B further enhances the stopband by adopting $\:{\mathrm{W}}_{\mathrm{1}}$ = 0.8 μm, $\:{\mathrm{W}}_{\mathrm{2}}\text{}$= 2.1 μm, $\:{\mathrm{W}}_{\mathrm{3}}$= 1.3 μm, and *P* = 530 nm, yielding Δ$\:\lambda\:$ ≈ 48 nm (Fig. 2 (a), green trace).

In both cases, we characterise the DBR families in transmission, by considering $\:\mathrm{N\:}$= 1000 uniform gratings periods.

Family C implements an air-gap DBR architecture (*W*_*1*_ =0) to further increase the stopband width by maximising the effective index contrast in the grating^22^.

Due to constraints on the minimal feature size, the narrowed width adopted in the DBR apodisation is $\:{W}_{1}=50{\hspace{0.17em}nm}$, after which the design transitions to air gaps between the SiN segments (Fig. S6 Supplementary Material Sect. 1).

The stopband behaviour is extracted from the transmission spectrum of the air-gap SC-FP characterised by $\:\mathrm{N}$= 1750, $\:{\mathrm{N}}_{\mathrm{T}}\mathrm{\:=250}$, *L* = 370$\:\:\mu m$, $W_{2} \: = \:2.3\:\upmu {\mathrm{m}}$, *P* = 526 nm, and DC = 70% (Fig. 2(b)).

This configuration enables the broadest operational bandwidth. The latter is estimated by evaluating the wavelength range over which the SC-FP transmission dips, as defined in^22^, are supported and exhibit a value of approximately $\:\Delta \lambda \: \approx 74\;{\mathrm{nm}}$.

Table 1 summarises the Δ$\:\lambda\:$ performance and the design parameters considered for the three DBR families.

**Table 1:** Experimental bandwidth among different DBR families.

DBR family	W_1_	W_2_	W_3_	$\:{\Delta\:}\lambda\:$	
	[µm]			[nm]	
A	1.3	2.5	1.3	DC = 40%	10
				DC = 50%	14.2
				DC = 70%	18.8
B	0.8	2.1	1.3	DC = 50%	48
C	0	2.3	1.3	DC = 50%	60*
				DC = 70%	74*

*For air-gap DBRs, the stopband is approximately estimated by considering the region in which are supported the SC-FP transmission dips, as carried out in^22^.

Furthermore, Table 2 provides a comprehensive review of photonic integrated Bragg gratings and DBRs from the literature, highlighting stopband widths, operating bands, core/substrate thicknesses, cladding configuration, and minimum feature sizes in order to compare with the devices presented in this work.

For instance, Ta₂O₅ rib-based DBRs on glass substrates immersed in water achieve $\:\varDelta\:\lambda\:$ ≈12 nm, near-UV bands (~ 850 nm) for label-free biosensing^25^. Triangular corrugation-shaped SiN DBRs (600 nm thick SiN on 1.4 μm SiO_2_ bottom cladding and 1.5 μm top cladding) exhibit a polarisation-independent 15 nm stopband in the O-band, with 40 dB rejection^26^. SOI strip-based gratings from^27^, realised on the 220 nm thick SOI platform, implement a single mode waveguide width of *W*_*1*_=500 nm and report a stopband ranging from 10 nm to 30 nm in the C-band, depending on air or silica cladding and corrugation width. SiN chirped spiral Bragg gratings demonstrate large group delay dispersion (around 1440 ps)^28^ and stopband of around 9.5 nm for 90 nm thick SiN, under SiO_2_ cladding, while SOI platform-based^29^ delivers $\:{\Delta\:}\lambda\:$ ≈15 nm with 50 ps group delay dispersion contribution.

The family B DBRs in this work, using 300 nm SiN with double tapering of both low- and high-index regions, deliver a broader $\:\varDelta\:\lambda\:$ ≈ 48 nm stopband thanks to the enhanced effective index contrast. By analysing the air-gap DBR architecture, our prior architectures, based on *W*_*1*_=1300 nm, *W*_*2*_=3000 nm in a 300 SiN platform achieved $\:\varDelta\:\lambda\:\:$>90 nm^22^.

This work introduces family C DBRs, which build on the air-gap design concept in^22^, to achieve $\:{\Delta\:}\lambda\:$ ≈ 74 nm stopband while realising a more compact footprint by reducing the grating high-index corrugation to $\:{W}_{2}=\mathrm{2300}$ nm.

**Table 2:** Comparative overview of stopband performance for various integrated DBRs.

Structure	Platform	Footprint [W×t]	Stopband$\:\boldsymbol{\Delta\:}\boldsymbol{\lambda\:}$	Ref.
RIB-based DBR	Dielectric Ta_2_O_5_ core, Water cladding, Substrate: Schott D263	Rib Core: W = 1000 nm Thickness: t = 160 nm Min. feature size: 40 nm	≈ 12 nm: UV-band	^25^
Triangular-shaped DBR	SiN core, 1.5 μm SiO_2_ cladding Substrate: 1.4 μm BOX	Core: W_1_= 600 nm, W_2_=1000 nm, Thickness: t = 600 nm, Min. feature size: 200 nm	≈ 15 nm, ER = 40 dB: O-band	^26^
DBR	Silicon core, air cladding Substrate: 2 μm BOX	Core: W_1_: 500 nm, W_2_: 820 nm, Thickness: t = 220 nm, Min. feature size: P/2 ≈ 162 nm	≈ 30 nm, ER *>* 30 dB C-band	^27^: Fig. 2.22
Air-gap DBRs	SiN core, air cladding Substrate: 3 μm BOX	Core: W_1_: 1300 nm, W_2_: 3000 nm, Thickness: t = 300 nm, Min. feature size: tip width ≈ 50 nm	≈ 90 nm: C-band	^22^
	SiN core, air cladding Substrate: 8 μm BOX	Core: W_1_: 1300 nm, W_2_: 2300 nm, Thickness: t = 300 nm, Min. feature size: tip width ≈ 50 nm	≈ 74 nm: C-band	This work
Chirped-DBRs	SiN on SOI core, SiO_2_ cladding	Core: W_1_: 2800 nm, W_2_: 3000 nm, Thickness: t = 90 nm, Min. feature size: 100 nm	≈ 9.5 nm, ER = 20/30 dB C-band, 1440 ps group delay	^28^
	SiO₂-on-Si rib core, Air cladding, Substrate: SiO_2_	Rib footprint: N.A., 5 mm length Min. feature size: N.A	≈ 15 nm: C-band, 50 ps group delay	^29^
DBR	SiN core, air cladding Substrate: 8 μm BOX	Core: W_1_: 800 nm, W_2_: 2100 nm, Thickness: t = 300 nm, Min. feature size: 150 nm	≈ 48 nm, ER = 30 dB C-band	This work

Broad spectral responses enhance DBR versatility for sensing, lasers, and telecom.

They enable multiplexing for multi-analyte detection across spectral regions, robustness to fabrication variations, and advanced multi-wavelength interrogation for improved accuracy.

## Characterisation and performance analysis

The frequency-dependent penetration length and phase response of the DBR introduce dispersion into the SC-FP cavity^15^. While this effect has been modelled, focusing on the prediction of the FSR response^9^ for both uniform and apodised gratings we experimentally demonstrate tailored integrated dispersion control across the C-band through systematic engineering of the DBR sections. In the optical frequencies domain ($\:\mathrm{f}$), the integrated dispersion $\:{\mathrm{D}}_{\mathrm{int,\:f}}$ (*µ*), is defined as:

1 $$\:{{D}_{int,f}\left(\mu\:\right)=f}_{\mu\:}\text{}-\left({f}_{0}\text{}+{D}_{1,f}\cdot\:\mu\:\right),$$

where $\:{f}_{0}$ and $\:{f}_{\mu\:}$ are the measured optical frequencies at the centre of the stopband and at the relative mode number $\mu$, while $\:{D}_{n,f}={\left.\frac{{\partial\:}^{n}{f}_{\mu\:}}{\partial\:{\mu\:}^{n}\:}\right|}_{\mu\:=0}$ are the *n*-th order dispersion coefficients, such that $\:{\mathrm{D}}_{\mathrm{1,\:f}}$ is the FSR at the centre of the stopband. We estimate the integrated dispersion $\:{\mathrm{D}}_{\mathrm{int,\:f}}\:\left({\mu}\right)$ by fitting the experimentally measured resonant frequencies.

$\:{f}_{\mu\:}$ with a cubic polynomial as:

2 $$\:{f}_{{\mu}}=\text{}{f}_{0}+{D}_{1,f}\cdot\:{\mu}+\frac{{D}_{2,f}}{2}\cdot\:{{\mu}}^{2}+\frac{{D}_{3,f}}{6}\cdot\:{{\mu}}^{3}.$$

**Fig. 3:**
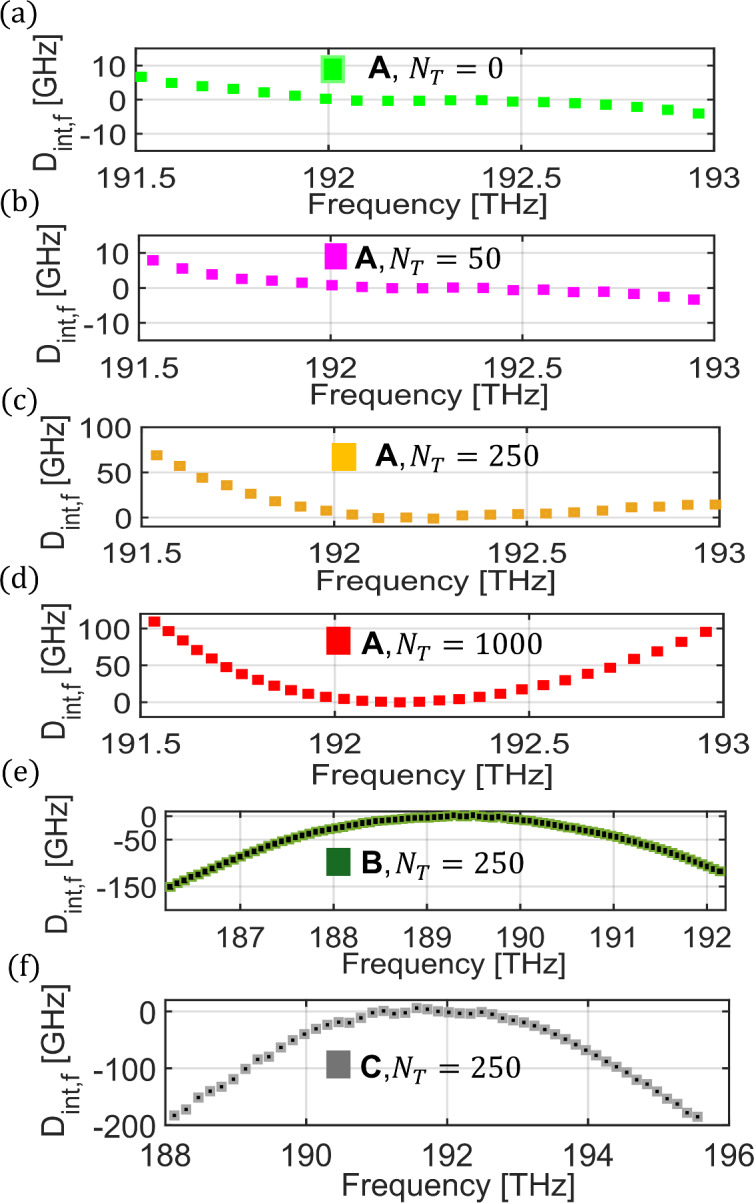
Dispersion control enabled by different DBR geometries. (a)-(d) Progressive transition from nearly flat to highly anomalous dispersion achieved by increasing the number of taper periods $\:{\mathrm{N}}_{\mathrm{T\:}}$, considering same family A DBR structure. Highly normal dispersion supported by broadband-based reflectors: (e) family B and (f) family C gratings.

Consequently, $\:{D}_{1,f}$, $\:{D}_{3,f}$, and $\:{D}_{3,f}$ are extracted from the fit coefficients. Figure 3 shows the integrated dispersion plotted as a function of optical frequency.

All the SC-FP resonators employ a total number of grating periods $\:\mathrm{N}+{\mathrm{N}}_{\mathrm{T}}\mathrm{\:=}$ 2000 to ensure maximum reflection within the stopband, with corrugation dimensions and period matching those of the corresponding DBR family.

For family A-based SC-FPs, we observe that increasing the number of apodised sections from $\:{\mathrm{N}}_{\mathrm{T}}\:\mathrm{=}\text{}$0 to $\:{\mathrm{N}}_{\mathrm{T}}\:\mathrm{=}$ 1000 drives a controlled transition from near-zero to strongly anomalous dispersion ($\:{\mathrm{D}}_{\mathrm{int,\:f}}\:\left(\mu \right)\:$> 100 GHz), when we fix *L* = 900 μm and *G* = 500 nm, as illustrated in Fig. 3(a–d).

Specifically, for a lower number of tapers $\:{\mathrm{N}}_{\mathrm{T}}\mathrm{\:=\:0}$ (Fig. 3(a)) and (Fig. 3(b)), evolves from $\:\approx\:\mathrm{10}$ GHz to $\:\approx\:-\mathrm{10}$ GHz as the resonance frequency shifts from 191.5 THz to 193 THz, remaining nearly flat at the bandgap centre and indicating a strongly suppressed second-order term with dominant third-order dispersion.

In contrast, for $\:{\mathrm{N}}_{\mathrm{T}}\mathrm{\:=}$250 and $\:{\mathrm{N}}_{\mathrm{T}}$= 1000, the curve becomes more parabolic with a pronounced second-order contribution (Fig. 3(c, d)).

At fixed cavity length and taper number $\:{\mathrm{N}}_{\mathrm{T}}\mathrm{\:=\:250}$, SC-FPs based on family B DBRs invert the dispersion sign compared to family A devices, yielding to a normal-dispersion regime with peak values of $\:D_{{{\mathrm{int}},f}} \left( f \right) \approx \: - {\mathrm{150}}\:{\mathrm{GHz}}$, as shown in Fig. 3(e). The normal dispersion regime is also observed for air-gap SC-FP, which use the same apodised grating length but a shorter cavity length (≈ 370 μm). For family C DBRs, $\:\left(\mathrm{P}\mathrm{=526\:nm,\:DC=70\%}\right)$ the air-gap SC-FP achieves larger normal dispersion, with peak $\:D_{{{\mathrm{int}},f}} \left( f \right) \approx \: - {\mathrm{190}}\:{\mathrm{GHz}}$ (Fig. 3(f)).

**Fig. 4:**
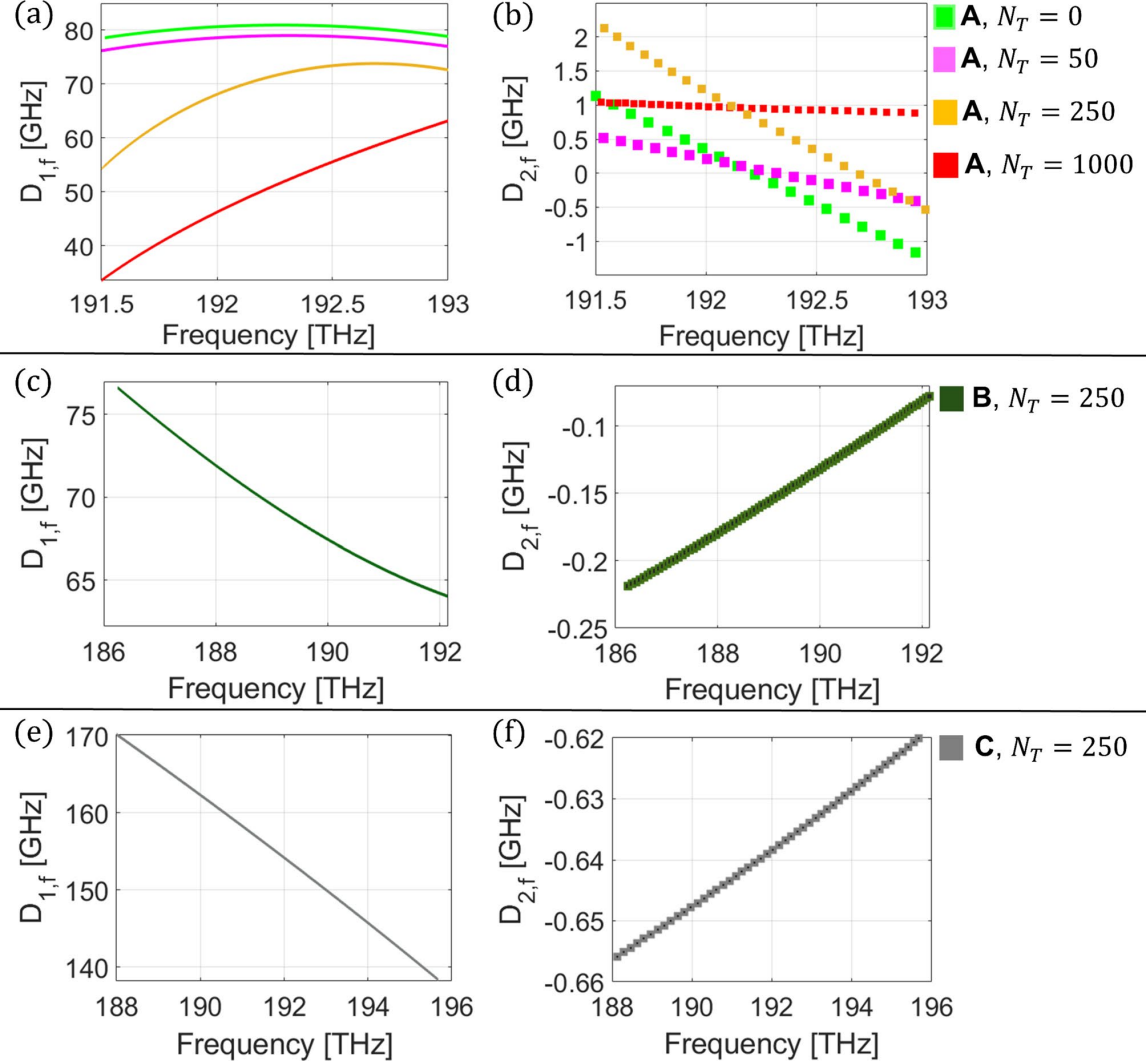
Fitted experimental first order (left side) and second order dispersion (right side) coefficients for: **a**–**b** family A, **c–d** family B, and **e**–**f** family C-based SC-FP resonators.

The dispersion properties of the SC-FP resonators are further analysed in Fig. 4 by plotting the fitted first-order coefficient $\:{D}_{1,f}$ and second-order coefficient $\:{D}_{2,f}$ as functions of optical frequency for the three resonator families. For family A devices, Fig. 4(a) shows that $\:{D}_{1,f}$ is almost flat for $\:{N}_{T}=0$ and $\:{N}_{T}=50$, indicating a nearly frequency-independent FSR of about 80 GHz and 75 GHz, respectively, and hence only very weak second-order dispersion.

In detail, $\:{D}_{2,f}$ exhibits very small values that cross from positive to negative around zero (Fig. 4(b)), while the magnitude of $\:{D}_{3,f}$ increases to about $\:-50$ MHz and $\:-130$ MHz for $\:{N}_{T}=50$ and $\:{N}_{T}=0$, respectively (as detailed in *Fig. S7* in the Supplementary Material Sect. 2).

When $\:{N}_{T}$ is increased to 250, $\:{D}_{1,f}$ starts to show a clear frequency dependence, and for $\:{N}_{T}=\mathrm{1000}$ it varies strongly from about 35 GHz to 62 GHz towards higher frequencies, yielding a strongly monotonic trend with a pronounced positive slope. This reflects a strictly positive second-order dispersion $\:{D}_{2,f}\approx\:1$ GHz with an almost vanishing $\:{D}_{3,f}\approx\:0$ MHz, fully consistent with the smooth quadratic integrated-dispersion envelope reported in Fig. 3.

For family B (Fig. 4 (c), (d)), and family C (Fig. 4(e), (f)) SC-FPs, the fitted $\:{D}_{1,f}$ shows a strong, nearly linear negative slope across the stopband, with average values of approximately $\:-\mathrm{0.15}$ GHz and $\:-\mathrm{0.64}$ GHz, respectively.

These results indicate that the FSR decreases monotonically with increasing optical frequency and that both resonators operate in a regime of pronounced normal second-order dispersion, confirming the broad, concave-down $\:{D}_{\mathrm{int},f}$ profiles in Fig. 3(e), (f).

**Fig. 5:**
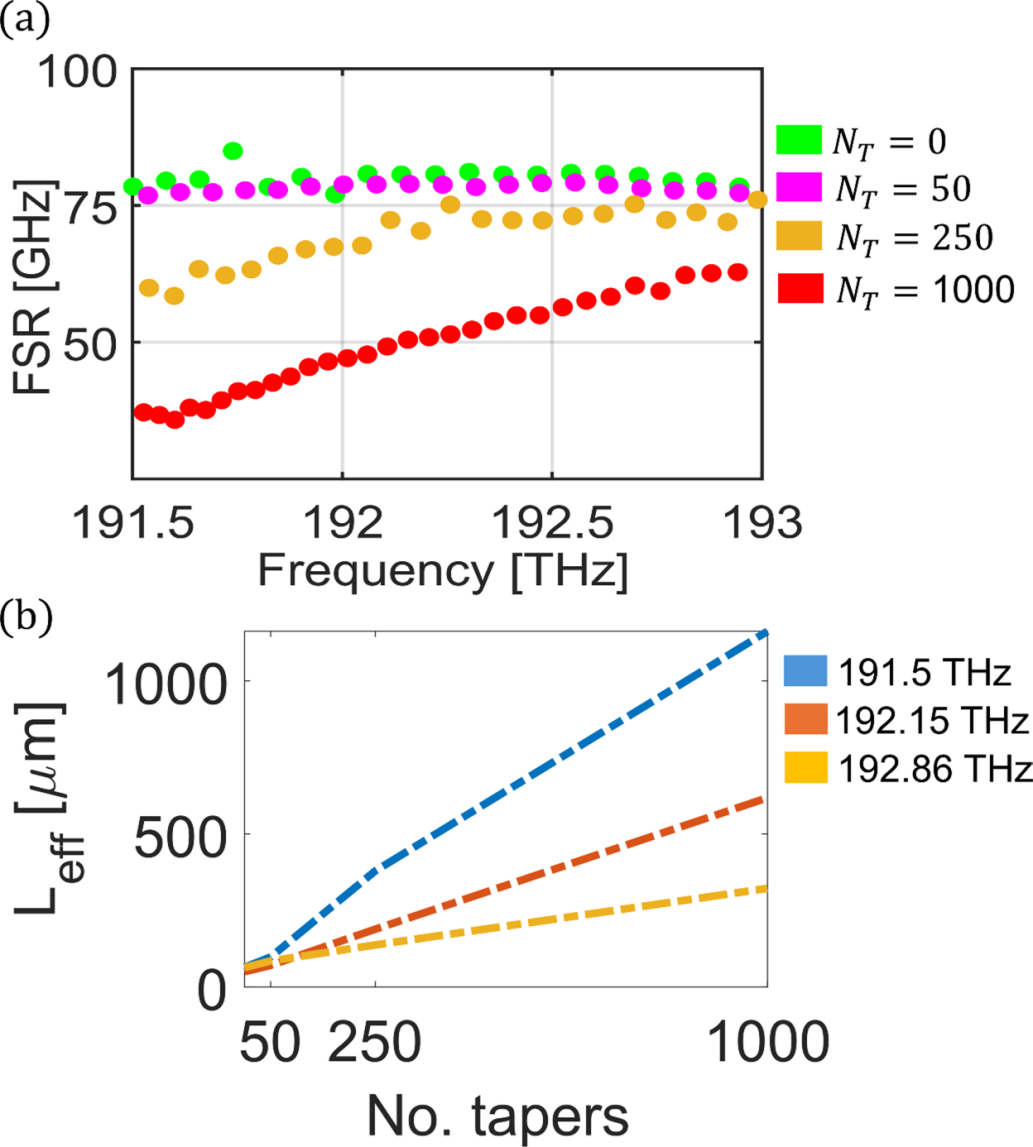
FSR response of SC-FPs based on family A DBRs with fixed cavity length $\:L=$900 $\:\mu\:\mathrm{m}$ and gap $\:G$=500 nm, for different apodised grating lengths $\:{N}_{T}$. **b** Corresponding experimentally extracted frequency-dependent penetration length $\:{L}_{\mathrm{e}\mathrm{f}\mathrm{f}}$, showing increased field extension at the stopband edges for longer DBR tapering sections. For $\:{N}_{T}\mathrm{=1000}$, $\:{L}_{\mathrm{e}\mathrm{f}\mathrm{f}}$ ≈ 500 $\:\mu\:\mathrm{m}$ at stopband centre and $\:{L}_{\mathrm{e}\mathrm{f}\mathrm{f}}$ ≈ 1000 $\:\mu\:\mathrm{m}$ at lower band edge.

The steepening of the first-order dispersion coefficient $\:{D}_{1,f}$ observed in family A-based SC-FP resonators with increasingly apodised DBR sections correlates with a larger penetration length at longer wavelengths (smaller optical frequencies). Moreover, the change in dispersion observed for longer tapered DBR sections is consistent with the measured resonator FSR response. Figure 5(a) shows the FSR response, in the frequency domain, while Fig. 5(b) reports the corresponding increase in penetration length toward smaller frequencies, experimentally extracted at the lower edge (191.5 THz), centre (192.15 THz), and upper edge (192.86 THz) of the stopband.

When the number of tapers is increased to $\:{N}_{T}=250$ and $\:{N}_{T}=1000$, the FSR shows a pronounced slope and strong frequency dependence. $\:{L}_{\mathrm{e}\mathrm{f}\mathrm{f}}$ which accounts for the additional penetration length beyond the physical geometric cavity length *L*=900 $\:{\mu}m$.

For longer tapering sections ($\:{N}_{T}=1000$), $\:{L}_{\mathrm{e}\mathrm{f}\mathrm{f}}$ nearly doubles between lower band edge and the stopband centre, indicating significant frequency-dependent penetration into the DBR mirrors, whereas its variation remains negligible for short tapers. Additional analysis on the effect of cavity length, in absence of apodisation, in terms of the FSR response are detailed in the *Supplementary Material*, Sect. 2, Fig. S8.

These results demonstrate that DBR engineering enables dispersion control, allowing the selection of either normal or anomalous dispersion at a target operating wavelength.

Beyond dispersion control, the SC-FP geometry enables co-optimisation of loaded quality factors ($\:{Q}_{l}$) and reflectivity through systematic variation of cavity length and coupling gap. In family A-based SC-FPs, we fix *L* = 900 μm and G = 600 nm, while introducing longer apodised gratings. The loaded Q-factor increases from $\:\mathrm{9.3}$×$\:{\mathrm{10}}^{\mathrm{4}}$ ($\:{\mathrm{N}}_{\mathrm{T}}$ = 0) up to 2.04$\:{\times}{\mathrm{10}}^{\mathrm{5}}$($\:{\mathrm{N}}_{\mathrm{T}}$ = 1000).

This enhancement is attributed both to the improved mode-matching ensured by the smooth transition between the cavity and the DBR sections, and the enhanced penetration length, as demonstrated in Fig. 5(b), that drastically extends the geometrical cavity length when

$\:{\mathrm{N}}_{\mathrm{T}}$ = 1000 apodised gratings are considered. However, the optimal Q-factor of ${\mathrm{Q}}_{{\mathrm{l}}} \: \approx$2.3 × 10⁵, that corresponds to a full-width at half maximum (FWHM) of ≈ 6.7 pm, is achieved when in the family A-based SC-FP is set $\:{\mathrm{N}}_{\mathrm{T}}\:\mathrm{=\:250}$, $\:\mathrm{G}$ = 400 nm, *L* = 900 μm while the coupler waveguide and the FP cavity width $\:{\mathrm{W}}_{\mathrm{4}}$ is increased from 1.3 μm to 1.8 μm.

The broadening of the resonant cavity waveguide reduces field overlap with the etched sidewalls and the relative scattering losses due to the roughness interaction, boosting the Q-factor.

SC-FP resonators incorporating family B gratings exhibit $\:{\mathrm{Q}}_{\mathrm{l}}$ $\: \approx$1.3 × 10⁵, for *L* = 900 μm, *G* = 600 nm and $\:{\mathrm{N}}_{\mathrm{T\:}}\mathrm{=\:250}$; while SC-FPs based on family C, characterised by $\:{\mathrm{N}}_{\mathrm{T}}\mathrm{\:=\:250}$, *L* = 370 μm *G* = 600 nm, *W*_*2*_ = 2300 nm, DC = 50%, yields a peak $\:{\mathrm{Q}}_{\mathrm{l}}$ $\approx$9.6 × 10^4^.

The abrupt discontinuity at the air-gap interfaces and relative mode-mismatch, introduces additional scattering and radiation loss, inducing an overall decreasing of Q-factor in the air-gap structures. The detailed Q-loaded analysis is reported in the Supplementary Material, Sect. 3, Fig. S9-S10.

These parameters result from a systematic optimisation of the cavity and grating geometry during the design process.

**Fig. 6:**
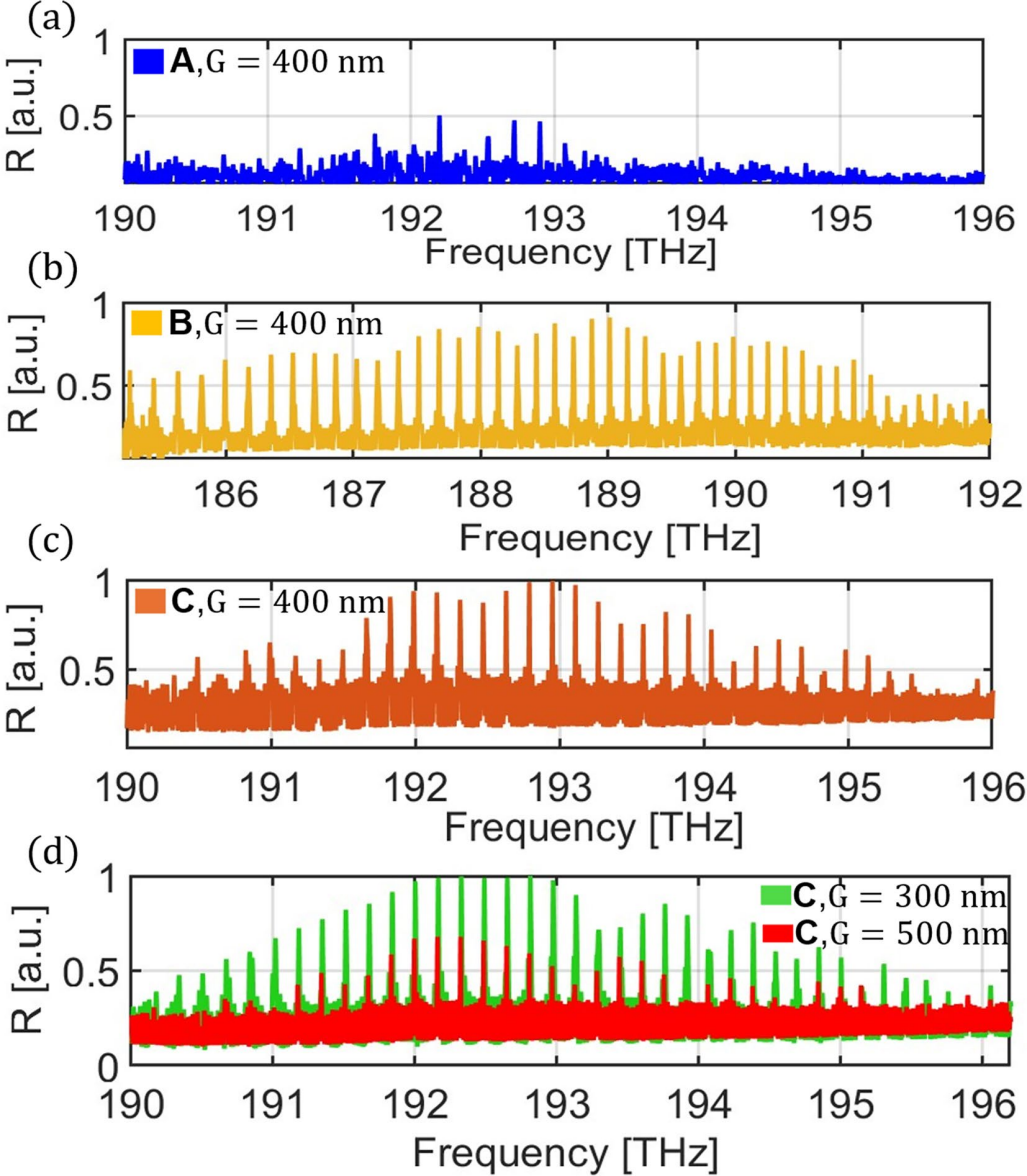
**a**-**c** Comparison between the normalised experimental reflectivity spectra of SC-FP based on family A, B, and C DBRs, for fixed $\:{N}_{T}=250$, $\:G$= 400 nm. **d** Air-gap SC-FP ($\:{N}_{T}$ = 250): increase in resonance peak intensity and extinction ratio with decreasing gap from 500 nm to 300 nm.

For laser integration, the effective peak reflectivity is a primary figure of merit that determines the suitability of SC-FP devices as resonant mirrors. This reflectivity is strongly governed by the underlying grating architecture and by the coupling gap engineered in the SC-FP.

The latter sets the resonator operating regime; in particular, operating in the over-coupled regime is advantageous in the SC-FP configuration, as it enables higher achievable reflectivity at the input waveguide port. For this purpose, we measured reflection spectra ($\:R)$ using a three-port fibre circulator for SC-FPs incorporating the three DBR families.

For family A and B-based resonators, we consider the same apodised grating periods *N*_*T*_ = 250, duty cycle DC = 50%, cavity length *L* = 300 μm and coupling gap *G* = 400 nm. The family C SC-FP configuration differs only by a shorter cavity length ($\:L=\mathrm{300}{\hspace{0.17em}}\mu\:\mathrm{m}$), while all other design parameters are kept identical.

The normalised reflection spectra of the three architectures are reported in Fig. 6(a–c). Enhancement in the on-resonance peak reflectivity ($\:{\mathrm{R}}_{\mathrm{peak}}$), combined with an increase in the maximum inter-peak extinction ratio $\:{\mathrm{ER}}_{\mathrm{inter-peak}}{ \approx }{\mathrm{R}}_{\mathrm{peak}}\mathrm{/}{\mathrm{N}}_{\mathrm{bg}}{\left.\text{}\right|}_{\mathrm{dB}}$, where $\:{\mathrm{N}}_{\mathrm{bg}}$ is the average background noise level, also due to parasitic FP arising from reflections at the chip cleaved facets, is observed in family B and family C-based SC-FPs.

Specifically, $\:{\mathrm{ER}}_{\mathrm{inter-peak}}$ reaches ≈ 6.35 dB for family B and ≈ 5.7 dB for family C, with a high density of well-defined reflection peaks over a 6-THz span, whereas SC-FPs integrating family A DBRs exhibit a degraded reflection response, with a maximum peak of ≈ 4.9 dB.

Among these structures, the coupled quality factor ($\:{\mathrm{Q}}_{c})$ remains invariant, while the improved behaviour can be attributed to the enhanced intrinsic quality factor ($\:{\mathrm{Q}}_{i})$ provided by the specific choice of DBR family. In this regard, family B gratings exhibit the best performance, as discussed in the Supplementary Material, Sect. 3, Fig. S11.

Moreover, the influence of the coupling gap on the reflectivity, for an identical grating architecture (family C SC-FP), is illustrated in Fig. 6(d).

As the gap decreases, $\:{\mathrm{ER}}_{\mathrm{inter}\mathrm{-}\mathrm{peak}}$ raises from $\approx$4.6 dB at $\:\mathrm{G}\mathrm{=500}\mathrm{\:nm}$ (red trace) to ≈ 6.77 dB at $\:\mathrm{G}\mathrm{=300}\mathrm{\:nm}$ (green trace), demonstrating improved reflectivity performance.

All resonators considered in the reflection analysis operate in the over-coupled regime ($\:{Q}_{i}>{Q}_{c}$), as confirmed by Figs. S12–S13 in the Supplementary Material, Sect. 3.

## Conclusion

We experimentally demonstrate highly tailored SC-FP resonators on a SiN platform through advanced Bragg grating mirror engineering. Systematic control of DBR geometry, including duty cycle, corrugation profile and depth, achieves broad optical stopbands exceeding 70 nm with air-gap SC-FP architecture, with potential to meet the bandwidth requirements of next-generation photonic systems for high-data-rate optical communications. Within a given SC-FP architecture, grating apodisation enables precise control over the dominant dispersion order in the cavity. Specifically, for short taper length the second-order term is strongly suppressed and third-order dispersion dominates, whereas increasing the number of tapered periods (*N*_*T*_ = 250 to *N*_*T*_ = 1000), steepens the FSR slope and yields a concave parabolic $\:{\mathrm{D}}_{\mathrm{int,\:f}}\:\left({\mu}\right)$ with peak magnitudes reaching 150 GHz in a strong anomalous regime, supporting applications such as dissipative Kerr soliton generation and parametric frequency comb systems. In contrast, for the same $\:{\mathrm{N}}_{\mathrm{T}}\mathrm{=250}$, switching to the broadband family B and family C DBRs drives the system into a strong normal-dispersion regime. Since the dispersion characteristics are predominantly governed by the DBR design, combining two different DBRs to define the SC-FP provides additional flexibility without requiring waveguide cross-section engineering. Co-optimisation of cavity length, coupling gap, and DBR family selection can support either high-Q resonances up to *Q*_*l*_ ≈ 2.3 × 10⁵ or maximised reflection peaks performance with $\:{\mathrm{ER}}_{\mathrm{inter-peak}}$≈6.77 dB.

These results establish SC-FP resonators as compact, fabrication-tolerant building blocks for scalable photonic integration, enabling advanced laser systems, nonlinear optics, and high-sensitivity sensing applications requiring precise, spectrally isolated high-Q resonances.

## Supplementary Information

Below is the link to the electronic supplementary material.

Supplementary Material 1

## Data Availability

Data underlying the results presented in this paper are not publicly available at this time but may be obtained from the authors upon reasonable request.
