# Supplementary material for: Broadband dispersion-engineered Bragg grating mirrors for integrated side-coupled Fabry–Pérot resonators
Source: Sci Rep. 2026 May 18;16:23132. doi: 10.1038/s41598-026-51041-9 (PMC13396758; doi:10.1038/s41598-026-51041-9)
Supplement: Supplementary file 1 — Supplementary Material 1 [file 41598_2026_51041_MOESM1_ESM.docx]

Broadband dispersion-engineered Bragg grating mirrors for integrated side-coupled Fabry-Pérot resonators: supplementary material document

Section 1. Fabrication details

The DBR and SC‑FP devices detailed in the manuscript were fabricated on LioniX SiN wafers using electron-beam lithography (EBL) with a negative‑tone hydrogen silsesquioxane (HSQ) resist directly patterned on the SiN surface. A 300 nm‑thick SiN core was deposited via low‑temperature plasma‑enhanced chemical vapor deposition (PECVD) on an 8 µm‑thick thermally grown SiO₂ bottom cladding over a 525 µm‑thick silicon substrate, with air acting as the top cladding.

Prior to lithography, the wafers were cleaned with acetone and isopropyl alcohol (IPA), then spin‑coated with an approximately 400 nm‑thick HSQ layer and soft‑baked to promote adhesion. Device layouts were exposed at 100 kV by raster‑scanning a focused electron beam, enabling higher resolution than positive‑resist trench exposures, and the unexposed HSQ was removed during development.


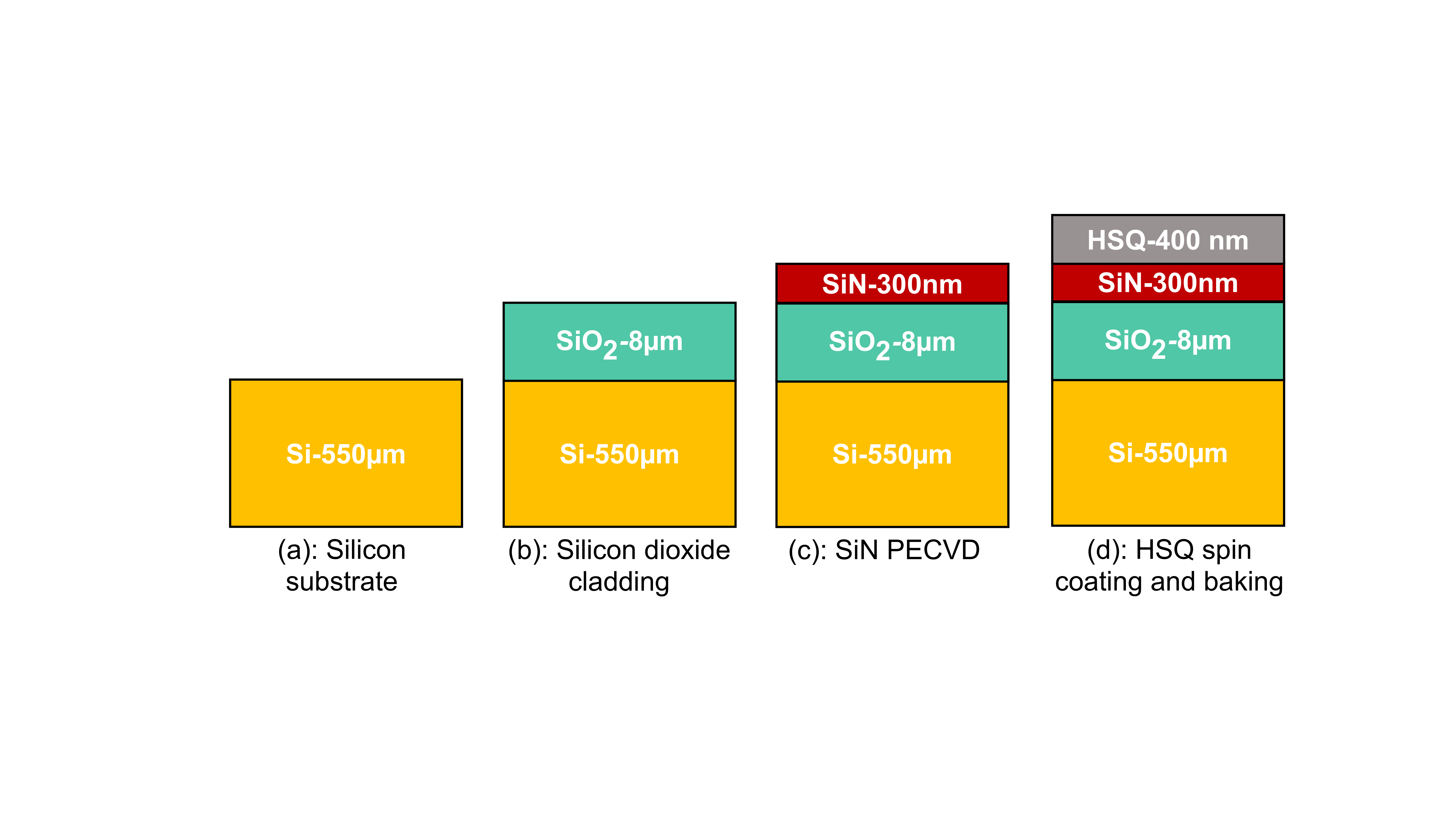


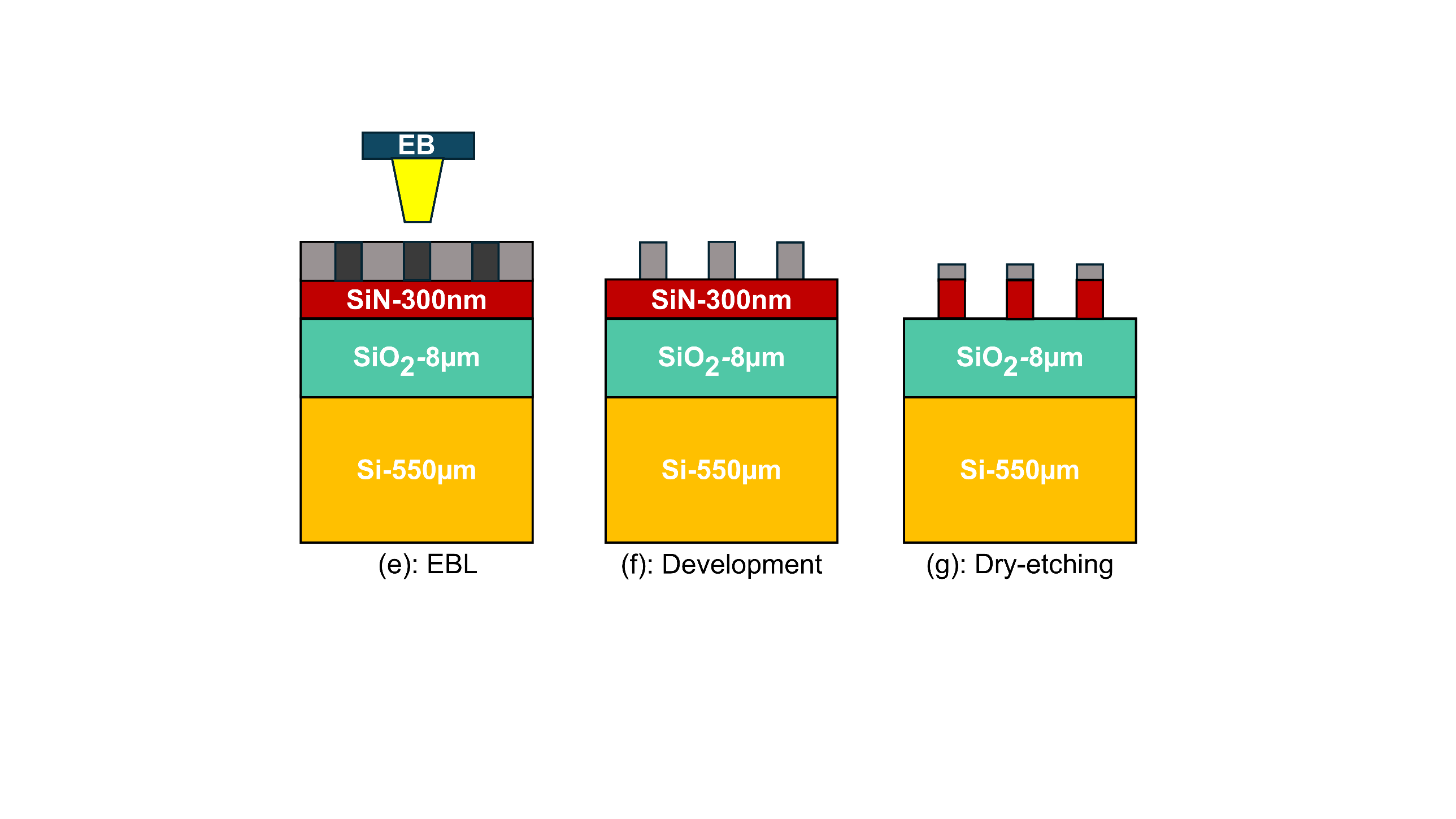


**Fig. S1:** Description of the PIC-fabrication flow for the realised DBR and SC-FP structures on a 300 nm thick SiN platform with 8 µm SiO_2_ bottom cladding and air as upper cladding.

The HSQ pattern was subsequently transferred into the Si₃N₄ layer by anisotropic inductively coupled plasma (ICP) dry etching using an O₂:CHF₃ (4:21) chemistry, which combines chemical etching with directional ion bombardment to achieve vertical sidewalls suitable for low‑loss waveguides.

From the initial 400 nm HSQ film, approximately 70 nm of residual mask material remained after the dry etching step. A wet‑etch removal using hydrofluoric acid (HF) was intentionally avoided in order not to expose the SiO₂ cladding to HF, thereby preventing additional scattering and absorption loss contributions that could degrade device performance while preserving the integrity of the photonic structures. The key fabrication steps are summarised in Fig. S1.

The ideal cross‑section of the single‑mode waveguide used in these structures is shown in Fig. S2(a), where perfectly vertical sidewalls (90°) are assumed in both the HSQ and SiN layers. In contrast, the scanning electron micrograph (SEM) in Fig. S2(b) reveals slight deviations from the nominal dimensions.

Specifically, the SiN core width designed as W_1_=1.3 µm reduces to approximately 1.26 µm, while W_2_ decreases from 2.5 µm to about 2.47 µm. Furthermore, the HSQ layer exhibits measured widths of roughly 1.114 µm and 2.33 µm, indicating a non‑ideal cross‑sectional profile and confirming that fabrication‑induced deviations from the target geometry are present.


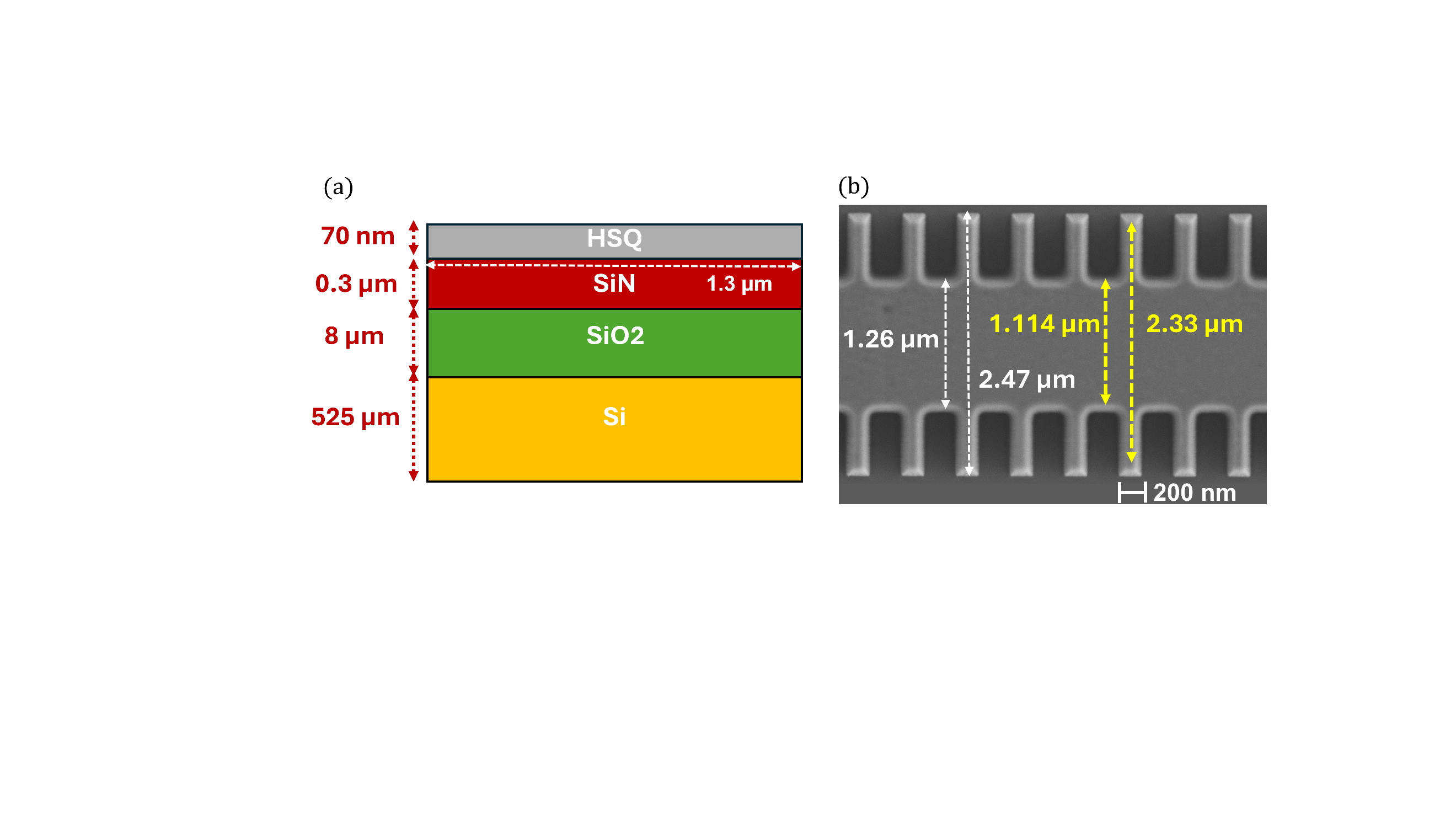


**Fig. S2:** Schematic cross-section of the ideal SC-FP in silicon nitride platform. (b) SEM images of the fabricated DBR device: indicating average etched dimensions for SiN (white) and HSQ layer (yellow).

Consequently, a more realistic cross-sectional schematic of the fabricated single-mode waveguide is shown in Fig. S3(a). Inspection of the SEM image in Fig. S3(b) confirms that the residual HSQ layer (approximately 70 nm) and the underlying ≈50 nm of SiN deviate from an ideal rectangular cross-section, instead forming a trapezoidal profile with non-vertical sidewalls. The sidewall features an initially steeper top angle that transitions into a shallower section before abruptly straightening into an almost vertical segment near the bottom of the core. This non-uniform profile, with sidewalls that are not perfectly vertical over the full waveguide height, is attributed to a non-optimised ICP dry-etching process.


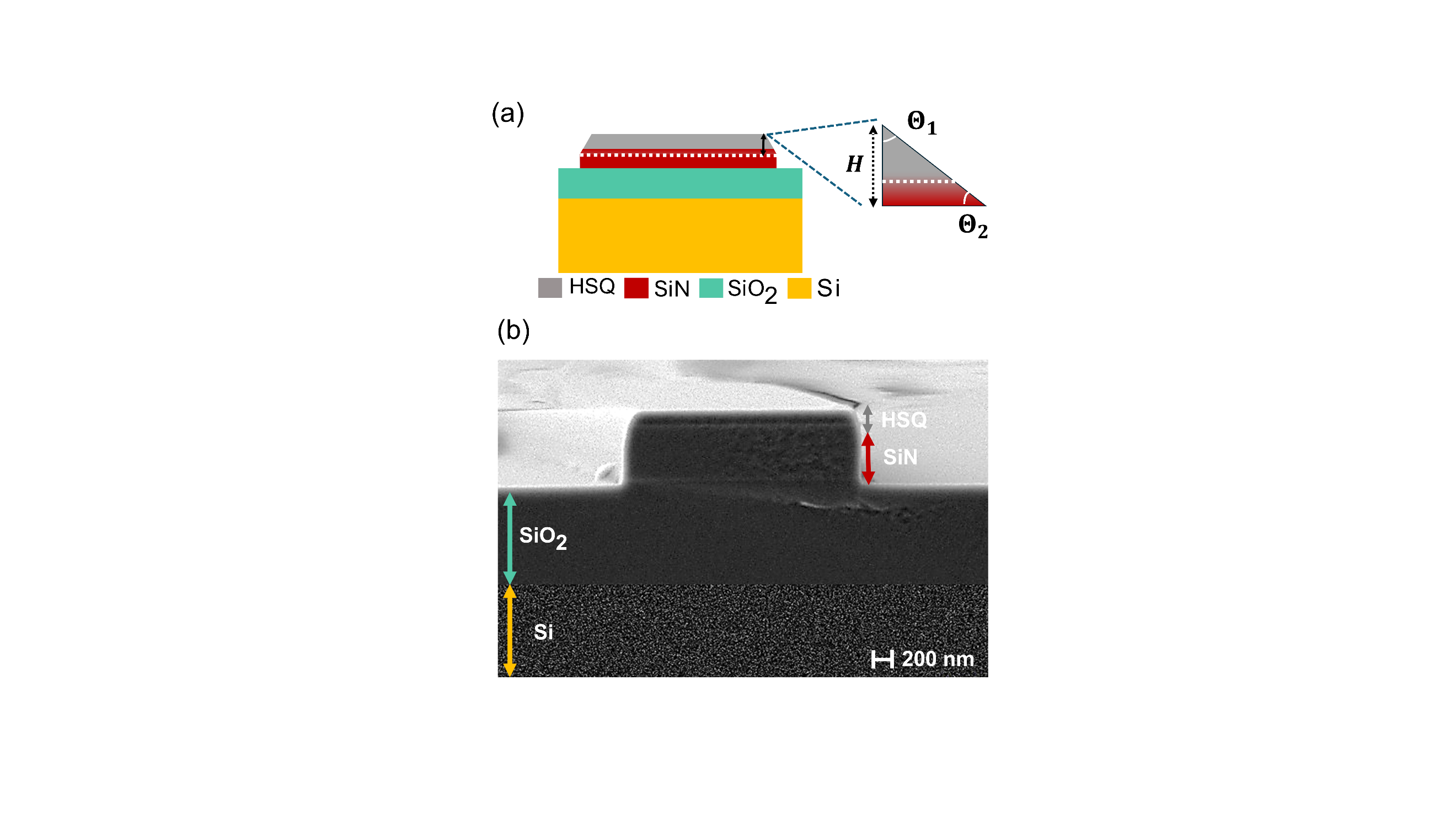


**Fig. S3:** (a) Schematic cross-section of the fabricated devices in the $y\text{–}z$ plane. Along the y-axis is defined the lateral waveguide width, while in z-axis are defined the different material thickness layers: $t_{HSQ}$=70 nm, $t_{SiN}$=300 nm, $t_{S{iO}_{2}}$=8 $\mu$m, and $t_{Si}$=525 $\mu$m. (b) SEM image of the single-mode waveguide cross-section.

Figures S4(a–c) show SEM images of the Family A DBRs, which feature a nominally rectangular corrugation and are designed for duty cycles of 40%, 50%, and 70%, respectively. By simple geometrical estimation, the corresponding effective duty cycles are reduced to approximately 33.5%, 44%, and 63%.

In particular, a target duty cycle of 50% is found, after fabrication, to shrink to an average value of about 44%. These deviations from the design values modify the effective index contrast and therefore impact the DBR bandwidth, the stopband center wavelength, and the grating coupling strength.

Figures S4(a–c) thus provide supporting fabrication details for Fig. 2(a) in the main manuscript.


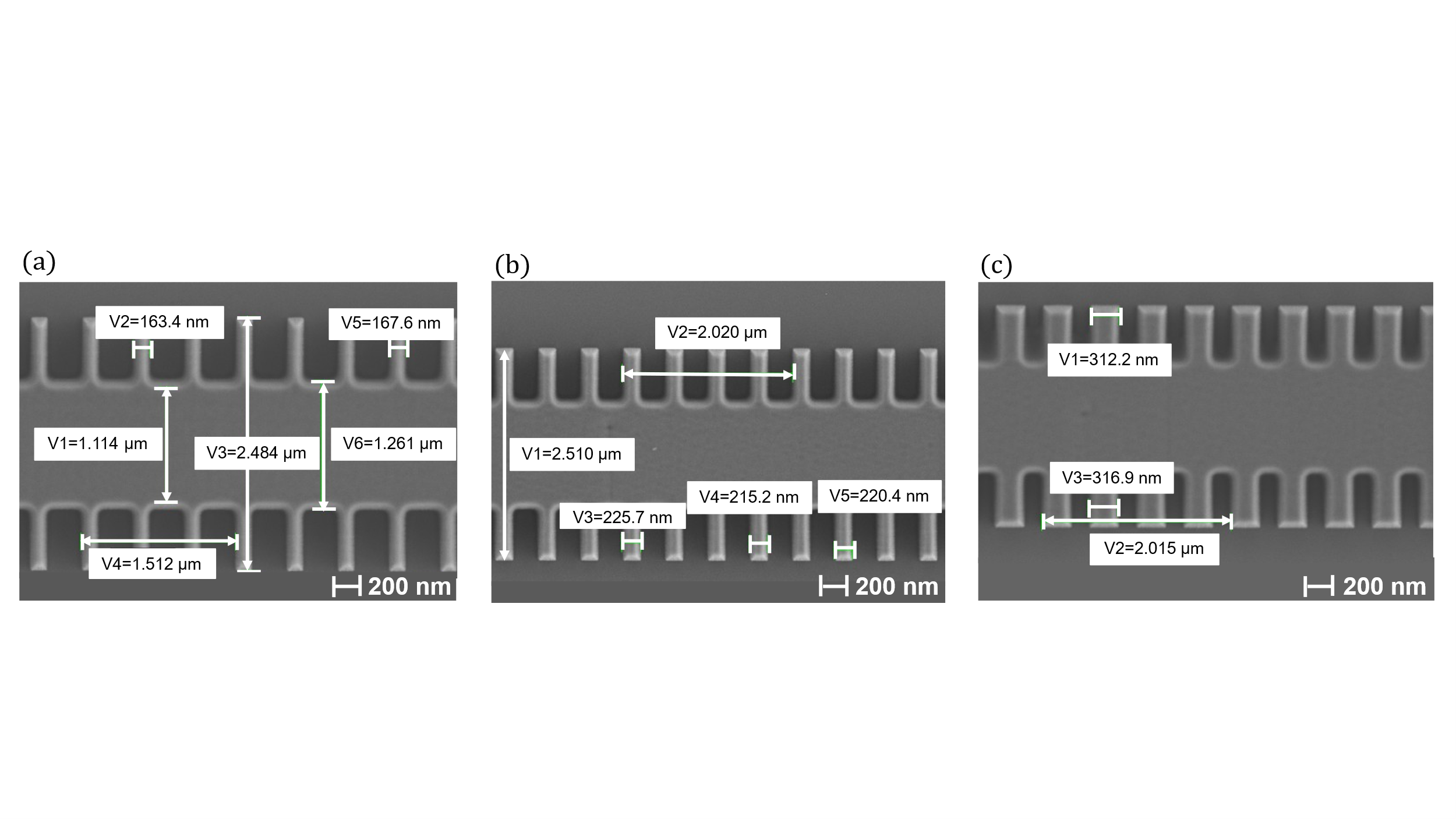


**Fig. S4:** SEM images of Family A-based DBR characterised by a rectangular corrugation shape and designed for duty cycles of (a) 40%, (b) 50%, and (c) 70%. Average corrugation width and etch dimension after fabrication process, are shown. By simple calculations, it can be estimated that the effective duty cycles result 33.5%, 44%, and 63%, respectively.

Figure S4 and S5 illustrate the schematic of the three different DBR families investigated in the manuscript and summarised in Table 1. Particularly, Fig. S4 (a) and (b) describe the Family A and B DBR mirrors, respectively, while Fig. S5 details on Family C DBR architecture highlighting the tapering of the waveguide width from W_3_ to W_1_, and the apodised DBR from W_3_ to W_2_.


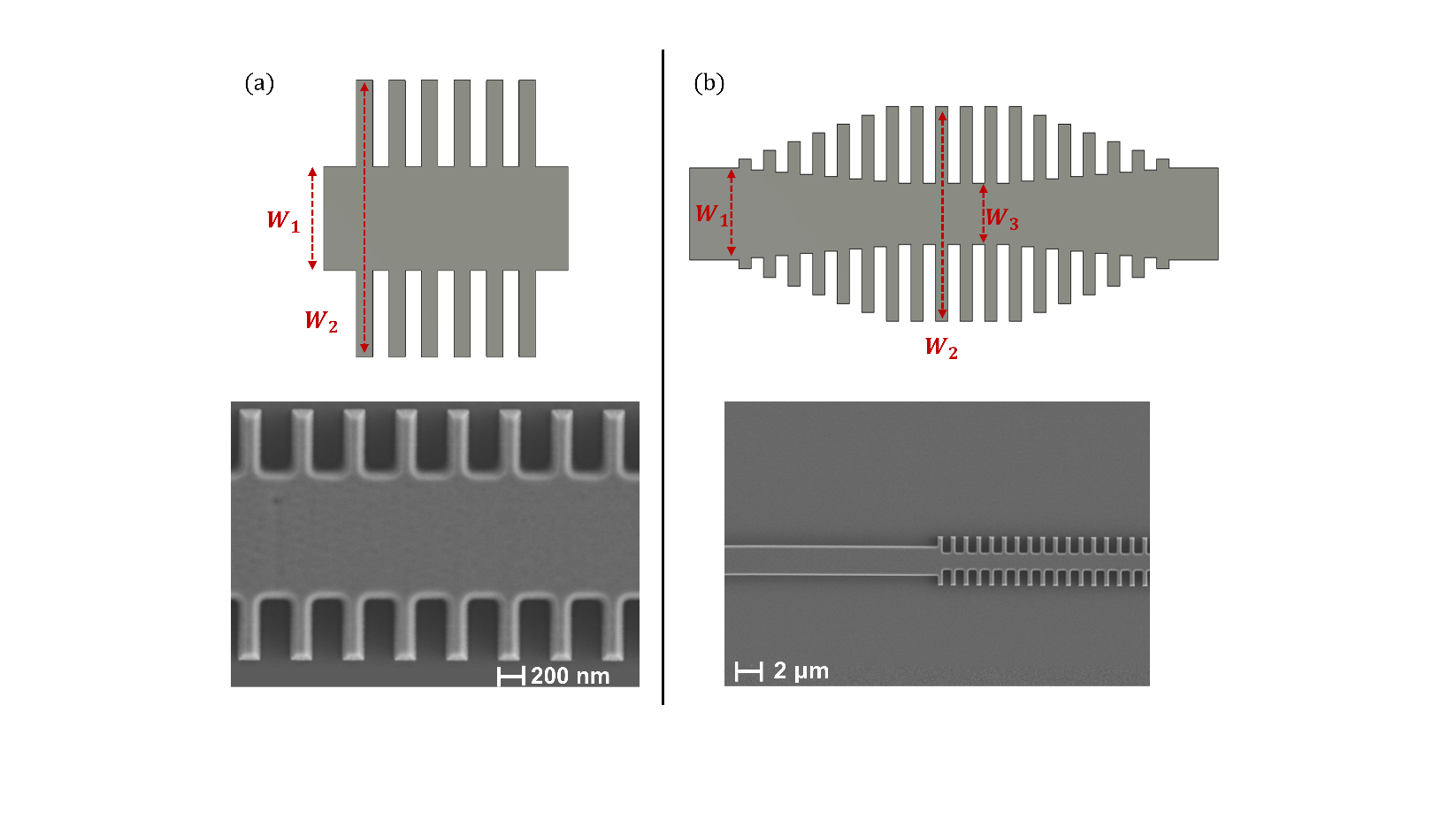


**Fig. S5:** Design schematic (top) and SEM image (bottom) for (a) family A and (b) family B DBR devices. It has been considered absence of corrugation tapering for simplicity.


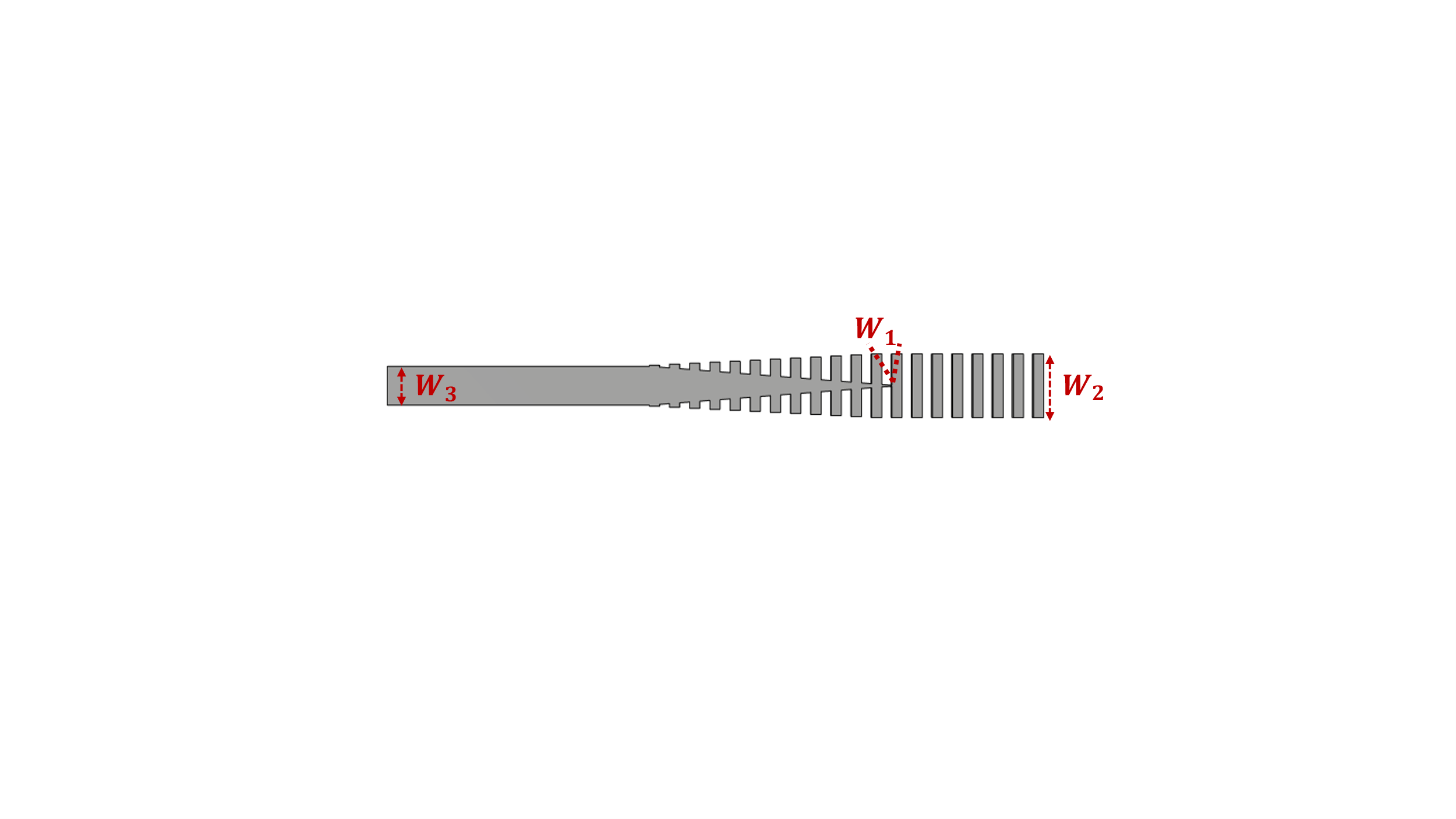


**Fig. S6:** Schematic of a family C DBR device, based on air-gap configuration.

Section 2. Dispersion engineering

Figure S7 supports the discussion of the integrated dispersion and fitted dispersion coefficients in the manuscript by highlighting the extracted third-order dispersion terms.

For the family-A SC-FPs, $D_{3,f}$ is approximately $-130$ MHz for $N_{T}=0$, $-50$ MHz for $N_{T}=50$, and close to 0 MHz for $N_{T}=1000$, showing a clear reduction in the magnitude of the third-order dispersion as the apodisation length increases

This trend is consistent with the quadratic integrated-dispersion envelope observed for the longer apodised gratings ($N_{T}=1000$), where a strictly positive second-order dispersion coefficient of about 1 GHz is accompanied by an almost negligible third-order term.

For the Family-B and Family-C configurations, the fitted $D_{3,f}$ values remain finite but small, on the order of 1.7 MHz and 0.7 MHz, respectively, indicating that third-order dispersion plays only a minor role in shaping the resonance spacing. In these cases, the dispersion response is dominated by the normal-dispersion regime, as reflected by the broad, concave-down $D_{\mathrm{int},f}(\nu)$ profiles with rapidly increasing slope away from the band center. Overall, these results distinguish Families B and C as strongly normally dispersive SC-FP configurations, in contrast to the more weakly dispersive and taper-tunable behavior of Family A.


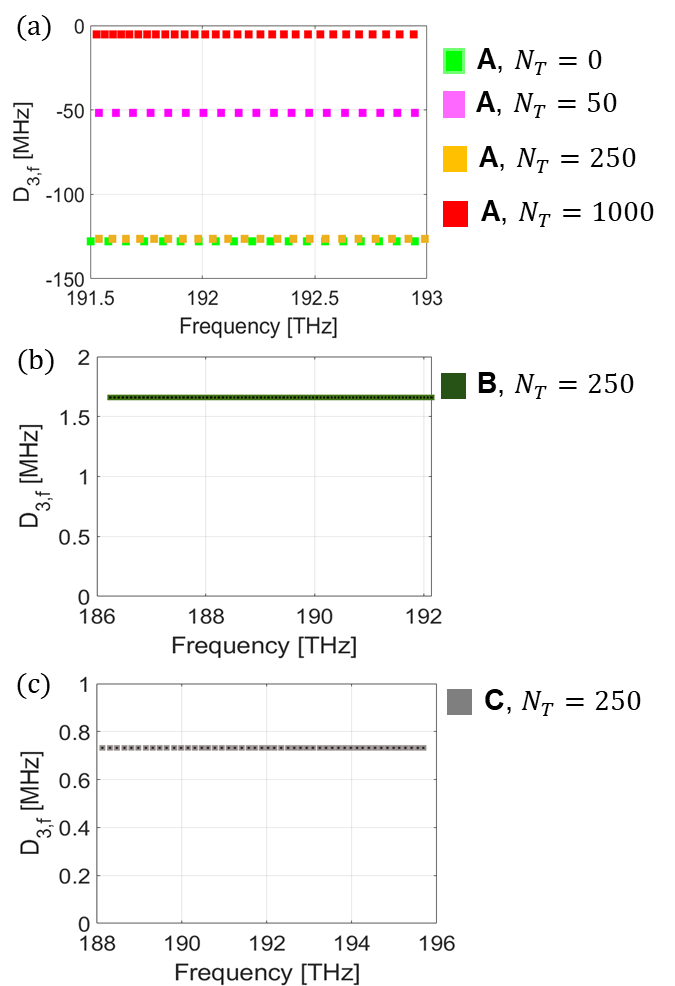


**Fig. S7:** Third order dispersion coefficient as function of the optical frequency for (a) Family A, (b) Family B, and (c) Family C-based SC-FPs in the manuscript.

Figure S8(a) presents the free spectral range response of SC-FP resonators integrating Family A DBRs, in absence of apodised sections, across a range of cavity lengths $L$.

The reported data demonstrate how increasing $L$ induces a progressive flattening of the FSR curve within the evaluated frequency band, yielding a more uniform mode spacing. Figure S5(b) explores the influence of different apodisation profiles on the FSR response for a fixed cavity length of $L=300 \mu$m and gap $\text{G}\text{=500}$ nm. The comparison among cubic (Mn=3), quadratic (Mn=2), and linear apodisation (Mn=1) profiles reveals that quadratic apodisation produces the least pronounced concavity in the FSR, exhibiting a more uniform spectral response compared to the linear and cubic cases.

The cubic and linear apodisation, conversely, results in the strongest concave parabolic FSR behaviour, indicating enhanced higher-order dispersion effects. These findings emphasise the critical role of apodisation design in engineering the dispersion within DBR-based SC-FP resonators, enabling tailored spectral profiles for optimised photonic device performance.


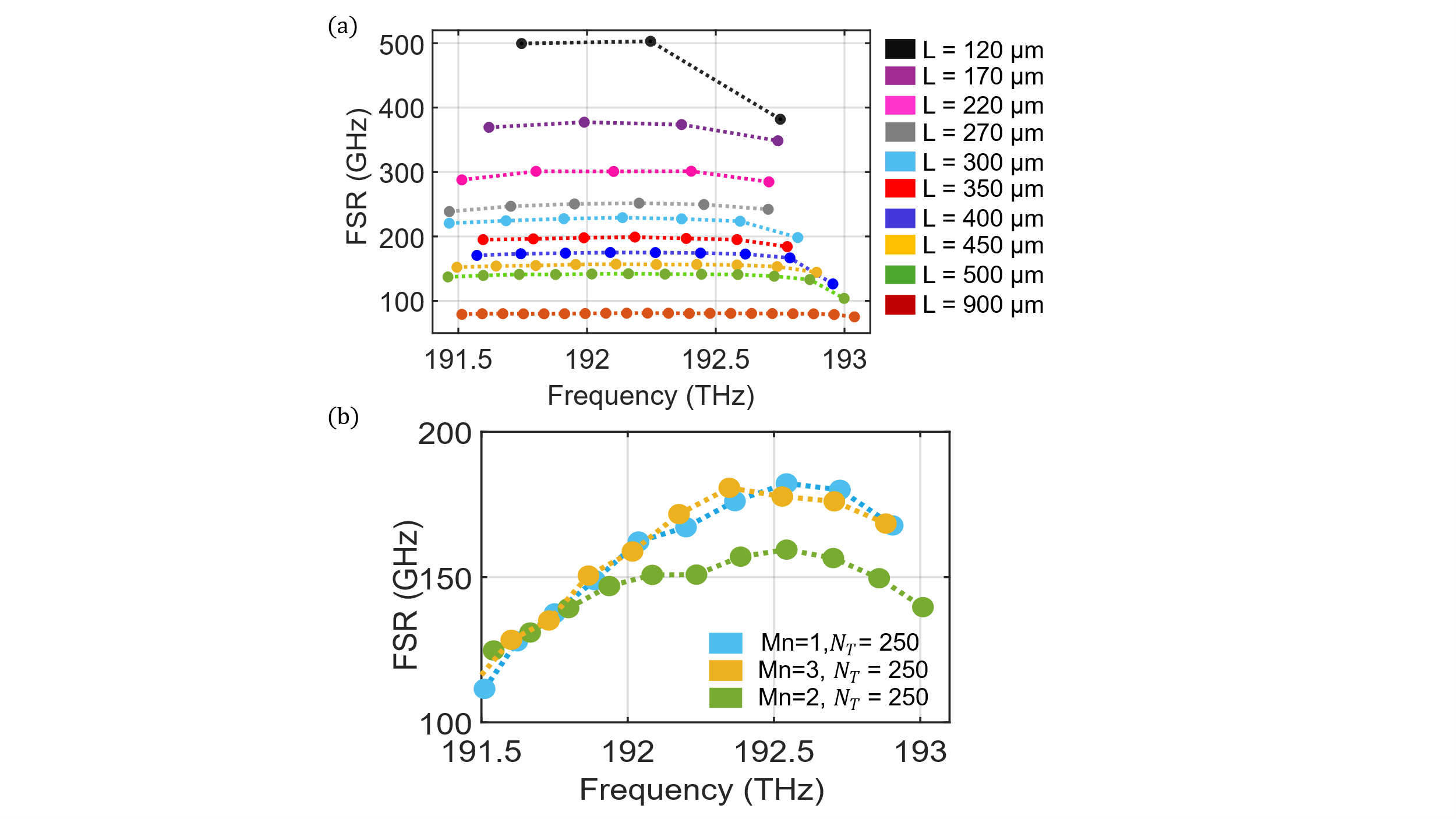


**Fig. S8:** (a) Calculated FSR versus frequency for SC-FP cavities of varying lengths, showing flattening of FSR with increasing L. (b) FSR response for linear, quadratic, and cubic apodisation at fixed length, emphasising the impact of grating shape on dispersion.

Section 3. Q-factor analysis

SC-FP resonances have been characterised with a tunable-laser source in continuous-sweep mode (sweep rate 1 nm s⁻¹, acquisition 100 kSample s⁻¹) and detected on a high-speed Germanium photodiode [New Focus, 800-1750 nm, Model 2033]. From these high-resolution experimental measurements, we fitted them according to the equations valid for a side-coupled resonator, described in [1], extracting loaded quality factor values.

Figure S9 shows the optimal loaded quality factors ($Q_{l}$) achieved for SC-FP resonators integrating Family A DBRs in two different variants. Peak  $Q_{l}$ value of 230,000 is obtained when the resonant cavity width is broadened from $W_{3}=1.3 \text{μ}\text{m}\text{ }$to $W_{4}=1.8 \mu$m, while the length is fixed to 900 $\mu$m and the gap to 400 nm. Moreover, the number of apodised DBR results$N_{T}$=250. The second architecture, that achieves $Q_{l}$≈200,000, is obtained when the resonant cavity width is kept constant to ${W_{4}=\text{ }W}_{3}=W_{1}=1.3 \text{μ}\text{m}$, the cavity length is equal to the first configuration, but the gap is set to 600 nm and the number of apodised DBR is increased to $N_{T}$=1000. In conclusion, the broadening of the resonant cavity width to 1.8 $\mu$m imposes some constraints in terms of the coupling gap. The bent waveguide, that composes the side-coupler, should be set to${W_{1}=W}_{4}$ to ensure the mode-matching between waveguide and cavity. This avoids further increments of the gap value over 400 nm to be able to observe the filter action of the DBRs in the SC-FPs. However, longer apodised sections can increase drastically the penetration length and ensure much higher loaded quality factors.


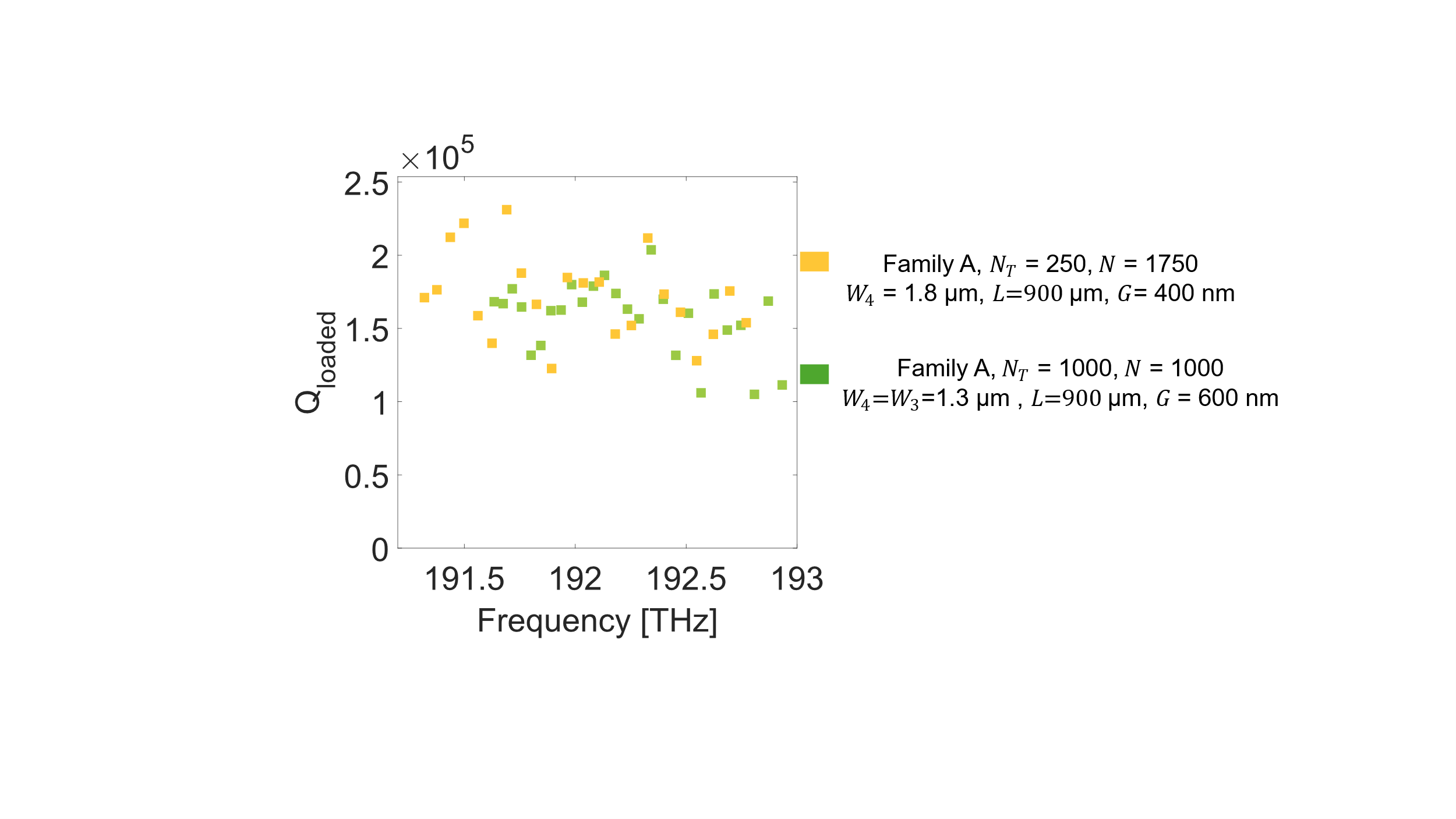


**Fig. S9:**  Loaded quality factors ($Q_{l}$) extracted from high-resolution measurements for Family A DBR SC-FPs, comparing the best performance achieved when considering $W_{4}=1.8 \mu$m (yellow trace) and $W_{4}=1.3 \mu$m.

A further analysis regards the loaded Q-factor obtained when SC-FP resonators incorporating family B and family C gratings are considered.

Specifically, Fig. S10(a) reports $Q_{l}$ as function of the frequency for family B-based SC-FP characterised by L=900 µm, G=600 nm, and $\text{N}_{\text{T}}\text{=250}$. The peak value is $Q_{l}$ $\approx$1.3×10⁵.

SC-FPs based on family C, characterised by $\text{N}_{\text{T}}\text{=250}$, L=370 µm G=600 nm, W=2.3 µm, DC=50%, yields to a maximum value of $Q_{l}$ $\approx$9.6×10^4^, as reported in Fig. S10(b).


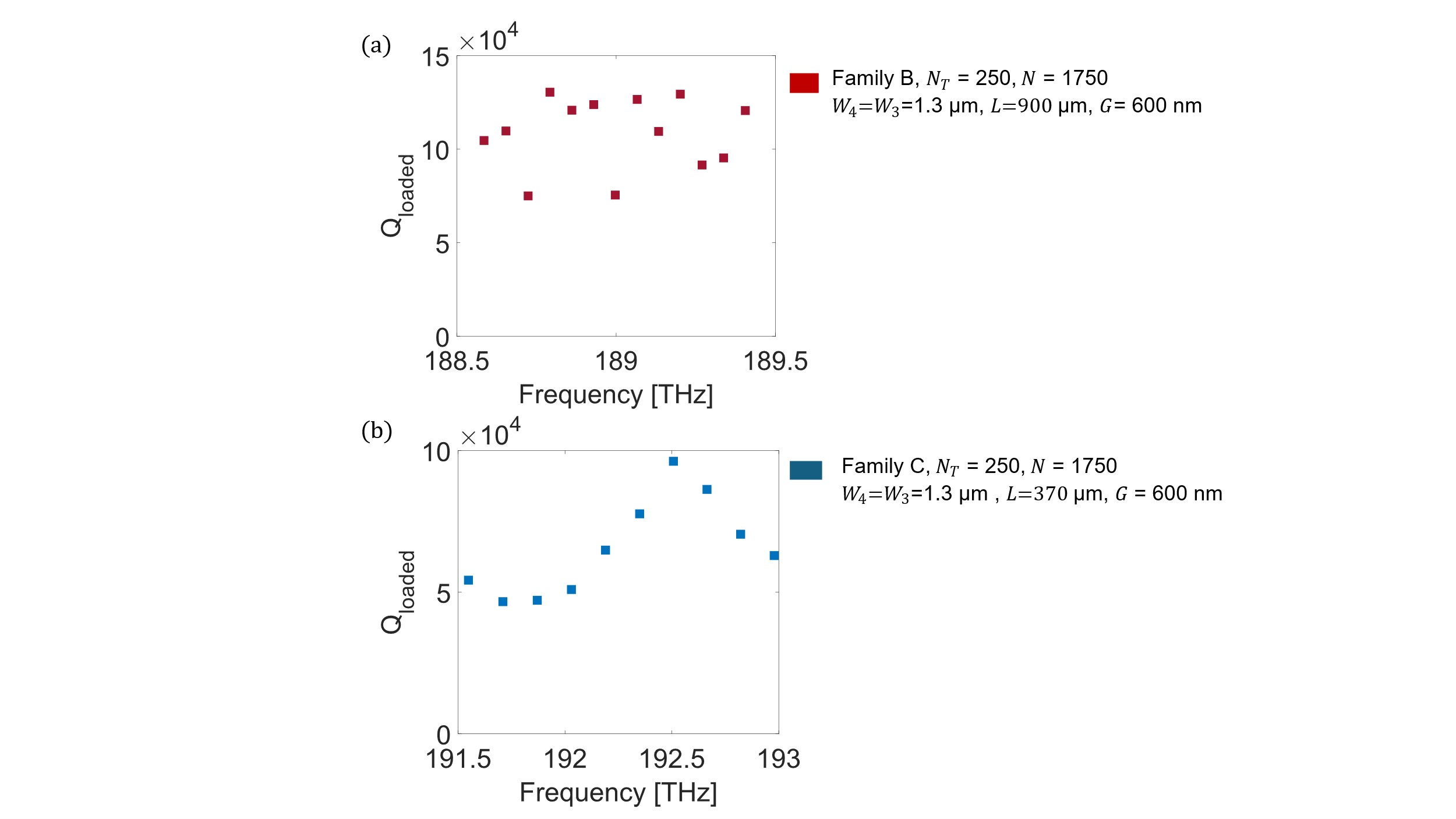


**Fig. S10:**  Loaded quality factor for SC‑FPs based on (a) Family‑B, and (b) Family‑C DBR.

Regarding the reflection peaks in Figs. 6(a–c) in the manuscript, here we report the different intrinsic quality factor ($Q_{\mathrm{intrinsic}}$) experimentally extracted for the resonant dips of the three configurations, as reported in Fig. S11.

The plot in Fig. S11 shows that $Q_{\mathrm{intrinsic}}$ is higher for the family B architecture ($Q_{\mathrm{intrinsic}}$>150000), while for the other devices it remains $Q_{\mathrm{intrinsic}}$<100000, which is consistent with the better response observed in the corresponding SC-FP reflection peaks. This indicates that, even for the same gap size (and consequently same coupled Q-factor), the resonator performance is strongly influenced by the family-dependent geometry and the associated intrinsic loss mechanisms.

*
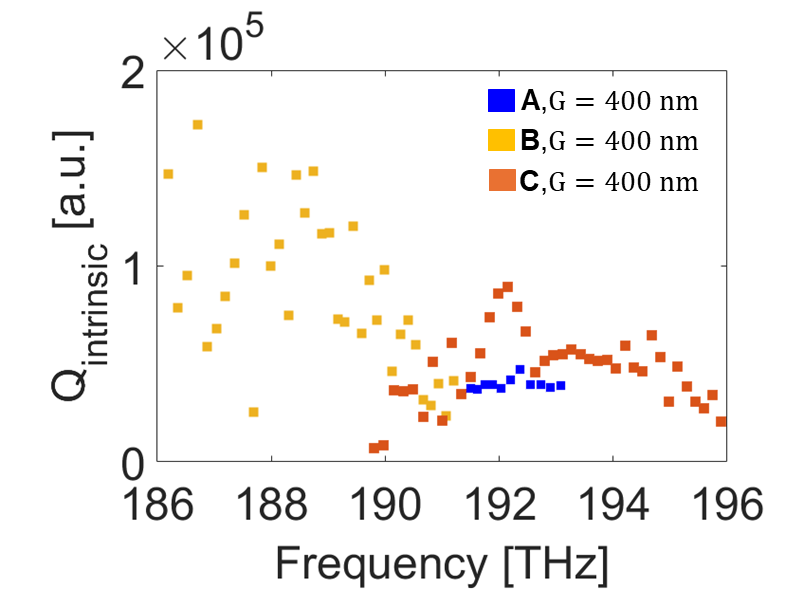
*

**Fig. S11:** Experimental intrinsic quality factor values for the three different SC-FP architectures: (blue) family A, (yellow) family B, and (orange) family C gratings sharing the same gap G=400 nm.

For the SC-FP resonators based on Family-C gratings, decreasing the coupling gap from larger to smaller values improves the performance of the on-resonance reflection peak, as shown in Fig. 6(d) in the manuscript.
To further support this behaviour, Figs. S12 and S13 report the intrinsic and coupled quality factors of the family C-based SC-FP devices for $G=600$ nm and $G=400$ nm, respectively. In both cases, $Q_{\mathrm{intrinsic}}>Q_{\mathrm{coupled}}$, with the effect being more evident for $G=400$ nm, confirming a clear overcoupled regime. This overcoupling is beneficial for achieving higher reflectivity in the SC-FP configuration and was deliberately selected.


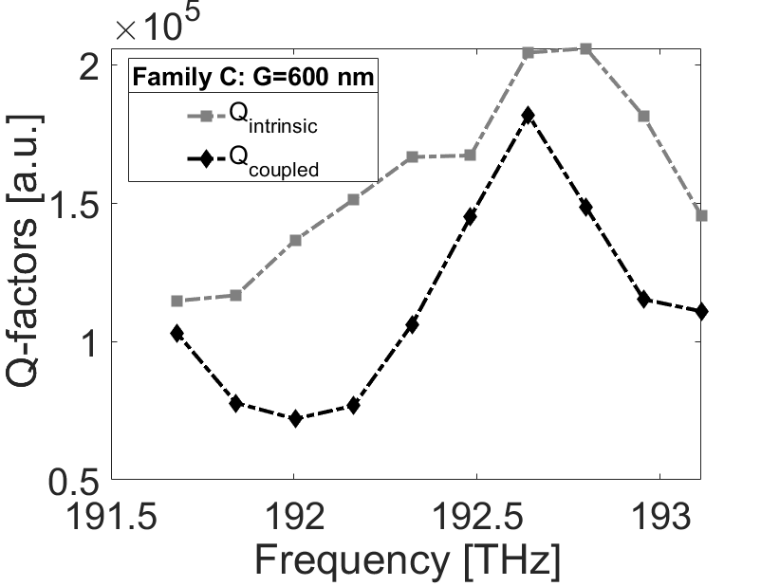


**Fig. S12:** Experimental intrinsic(grey) and coupled(black) quality factor values for SC-FP based on family C DBR with G=600 nm.


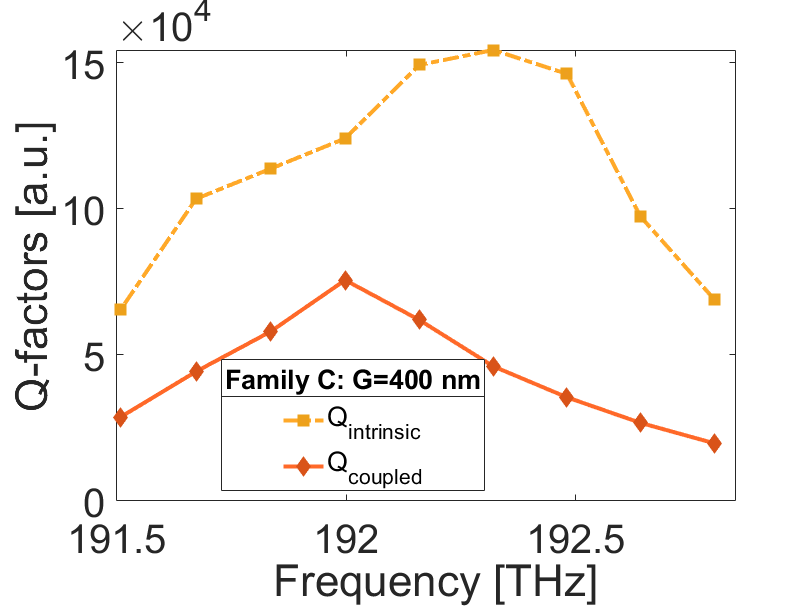


**Fig. S13:** Experimental intrinsic(yellow) and coupled(orange) quality factor values for SC-FP based on family C DBR with G=400 nm.

References

1. Xu Y, Li Y, Lee RK, Yariv A. Scattering-theory analysis of waveguide-resonator coupling. Phys Rev E Stat Phys Plasmas Fluids Relat Interdiscip Topics. 2000 Nov;62(5 Pt B):7389-404.
